# From Enrichment to Fate: Transport, Transformation, and Fate of Micro- and Nanoplastics in Marine Environments

**DOI:** 10.3390/toxics14020120

**Published:** 2026-01-27

**Authors:** Wei Ma, Xinjie Liang, Changling Ding, Yingying Ye, Jiji Li

**Affiliations:** 1 Research Centre for Indian Ocean Ecosystem, Tianjin University of Science and Technology, Tianjin 300457, China; mawei2030@huaihejg.mee.gov.cn; 2 Huaihe River Basin Eco-Environmental Monitoring and Scientific Research Center, Bengbu 233001, China; 3 National Engineering Research Center for Marine Aquaculture, Zhejiang Ocean University, Zhoushan 316022, China; liangxinjie@zjou.edu.cn (X.L.); yeyy@zjou.edu.cn (Y.Y.)

**Keywords:** microplastics, nanoplastics, ecotoxicology, degradation, biodistribution

## Abstract

With the increasing detection of micro- and nanoplastics (MNPs) in marine environments and the expanding body of related research, their environmental behavior and ecological effects have become central topics in marine environmental science. This review addresses the growing concern over MNP pollution in the marine realm, encompassing their primary sources, spatial accumulation and distribution, environmental transport and transformation dynamics, and ecotoxicological effects on marine organisms and ecosystems, as well as the ecological risks they pose within key habitats such as seagrass beds and coral reefs. We synthesize evidence on the biological impacts of MNPs, including oxidative stress, tissue accumulation, metabolic disturbances, and immune impairment, as well as the heightened risk of pathogen transmission facilitated by the so-called “Plastisphere”. Moreover, we explore the potential implications of MNP exposure on oceanic carbon cycling and net primary productivity. The reviewed literature suggests that MNPs are capable of long-range transport and progressive fragmentation into ultrafine particles, which are readily ingested and retained by a wide array of marine organisms, subsequently inducing toxicological effects and compromising both organismal health and ecological integrity. Such disturbances may undermine critical ecosystem services, including carbon sequestration capacity and food web stability. Finally, based on the current research landscape, we outline future research priorities: improving environmental detection and toxicological evaluation of MNPs, elucidating their long-term effects at the ecosystem scale, and investigating their interactions with co-occurring pollutants under complex, multi-stressor scenarios. These efforts are essential to support science-based assessment and effective management strategies for marine MNP pollution.

## 1. Introduction

Plastics are versatile and durable materials that are widely used across industries such as packaging, construction, textiles, and electronics. Since the 1960s, global plastic production has surged, with an estimated 33 billion additional tons being projected by 2050 [1]. The proliferation of single-use plastics, especially in food and beverage packaging, has significantly escalated plastic waste generation. Although disposal methods include landfilling, incineration, and recycling, a substantial portion still reaches aquatic environments through littering, wastewater discharge, surface runoff, landfill leachate, and illegal dumping [2,3]. The persistent increase in plastic use has led to widespread leakage and accumulation in marine systems, particularly in oceanic convergence zones such as the Pacific gyres [4]. Over five trillion plastic particles are estimated to be floating at the ocean surface, with additional debris accumulating on the seafloor [4]. The vast majority of marine plastic debris is categorized as either “microplastics (MPs)” or “nanoplastics (NPs)”. MNPs have emerged as some of the most ubiquitous marine pollutants, with their defining characteristic being their size. Specifically, MPs are defined as particles smaller than 5 mm, while NPs are those smaller than 1 μm [5], collectively referred to as MNPs. The size range that is used to define NPs varies among studies. Some reports apply a more restrictive definition (e.g., NPs ≤ 100 nm), whereas others consider a broader size range, extending the upper limit to 1 μm. As this review seeks to integrate available evidence on the sources, accumulation, and environmental fate of MNPs in marine environments, we adopt the broader definition herein, in which NPs include particles up to 1 μm. The pervasive presence of MNPs is driven not only by excessive plastic usage but also by their remarkable resistance to degradation, which contributes to their environmental persistence. MNPs are now considered among the most critical emerging contaminants in marine ecosystems due to their stability and ecological risk potential [6]. Once released into the ocean, MPs are nearly impossible to remove and have been detected in remote regions, including polar ice, the deep sea, and isolated islands [7]. Compared with microplastics, nanoplastics may potentially pose greater toxicological risks, as suggested primarily by laboratory studies and mechanistic considerations [8]; however, in situ evidence and environmentally realistic exposure–response relationships remain limited. Plastic debris exerts both immediate and long-term adverse effects on marine organisms, coastal esthetics, tourism, and human health [9]. Their environmental persistence further exacerbates ecological threats to marine ecosystems [10].

MNPs in the environment primarily originate from land-based sources, with an estimated 80% of the pollution stemming from urban runoff, industrial discharges, and agricultural activities, while only around 20% is directly attributed to marine-based sources [11]. Plastic debris and MNP-laden wastewater enter marine ecosystems predominantly through rivers, sewage systems, and other hydrological pathways [12]. Additionally, atmospheric transport represents a critical cross-media migration route; MNP particles can be dispersed into the atmosphere via wind and have been detected in precipitation and polar ice cores [13]. Common atmospheric sources of MNPs include textile fiber abrasion, tire wear particles, urban dust, and waste incineration emissions [14]. Other important emission sources include sewage sludge from wastewater treatment plants, residual agricultural plastic films, and organic fertilizers [15]. These particles can be transported via surface runoff into transitional environments such as estuarine wetlands, which act as potential accumulation hotspots [16]. Once introduced into the marine environment, MNPs often settle into deep-sea sediments, highlighting their potential for long-term environmental persistence [17]. The complexity of MNP transport, spanning multiple pathways and environmental media, has resulted in their widespread global distribution, including in remote regions with minimal anthropogenic influence. In marine ecosystems, MNPs engage in intricate physical, chemical, and biological interactions with surrounding media and coexisting pollutants. Due to their high surface area and hydrophobic characteristics, MNPs are prone to adsorbing toxic substances such as persistent organic pollutants (POPs) and heavy metals [18], effectively functioning as vectors for contaminant transport—an effect that is commonly referred to as “plastics carrying toxins”. These contaminant-laden particles may be ingested by marine organisms and transferred through trophic levels, ultimately leading to bioaccumulation in higher-order predators [19]. Furthermore, the surfaces of MNPs often support the formation of a specialized microbial biofilm known as the “Plastisphere”, which harbors diverse microbial communities, including pathogenic bacteria and antibiotic resistance genes. This phenomenon poses heightened risks to both marine ecosystem integrity and public health [20,21].

Interactions between MNPs and marine organisms primarily occur through ingestion and biological uptake. A growing body of research indicates that a diverse array of marine species, including plankton, bivalves, fish, and seabirds, can ingest MPs. These particles may cause mechanical injury, obstruct nutrient absorption, and trigger physiological responses such as inflammation and oxidative stress [22,23]. Furthermore, monomers and chemical additives that are embedded in MNPs can be released within the body, leading to endocrine disruption, immunosuppression, reproductive and developmental impairments, and an increased risk of carcinogenesis. Compared to MPs, NPs exhibit significantly higher bioavailability because of their ability to traverse cell membranes and migrate throughout the body. Experimental studies in animals have shown that NPs can accumulate in vital organs such as the liver, spleen, lungs, and kidneys. They can even cross the blood–brain barrier, thereby posing serious risks to neurological health [24]. Despite growing interest in marine MP pollution, our understanding of the environmental fate and biological impacts of MNPs remains limited. One major challenge is the lack of sensitive and reliable analytical methods that are capable of detecting NPs at environmentally relevant concentrations and spatial resolutions, making ecological exposure and toxicity assessments highly uncertain [25]. Moreover, the degradation and transformation pathways of MPs in marine systems are still poorly elucidated [26]. Although deep-sea sediments are generally considered a major sink for MNPs, recent studies have shown that NPs smaller than 10 μm may remain suspended in deep ocean waters for extended periods via the biological pump, often in association with zooplankton fecal pellets. In addition, MPs that are released during the melting of polar sea ice may re-enter marine circulation through an interconnected ice–ocean–atmosphere system [27]. While microbially mediated plastic degradation has shown promise under controlled laboratory conditions [28], its effectiveness in marine environments remains uncertain due to constraints such as low temperatures, high salinity, and nutrient-poor conditions.

Current research on MNPs is characterized by pronounced methodological heterogeneity and a lack of standardized data, leading to substantial uncertainty in both regional- and global-scale risk assessments and further widening the gap between laboratory simulations and real environmental conditions. In particular, studies on the toxicological effects of NPs face significant challenges: although conventional endpoint-driven toxicological assays—such as acute and chronic apical endpoints (e.g., survival, growth, and reproduction) and routine biomarkers of oxidative stress and inflammation—can be informative for hazard screening, when used alone, they are often insufficient to effectively track particles’ fate following NP exposure (including aggregation or corona formation, biodistribution, and translocation) or to establish clear links with downstream pathway-level mechanisms. These limitations are especially pronounced under environmentally relevant low exposure concentrations and highly heterogeneous particle properties. Together, these constraints constitute a critical knowledge gap in current toxicological research on NPs [29]. Moreover, the combined effects of MNPs with other pollutants, their transformation processes within organisms, and their trophic transfer patterns through food webs remain insufficiently explored. In summary, scientific understanding of MP pollution, which is an emerging and complex environmental issue, remains limited. There is an urgent need for more systematic, standardized, and environmentally relevant studies to address current knowledge gaps and provide theoretical and technical foundations for future pollution control, risk assessment, and ecological safety strategies. This review aims to systematically examine the sources and transport pathways, biological interactions, and ultimate fate of MNPs in marine environments, and to assess their potential impacts on ecosystem stability and organismal health. Specifically, it will (1) analyze the spatiotemporal distribution and physicochemical properties of MNPs and clarify their transformation and migration processes across multiple environmental media; (2) investigate the interaction mechanisms between MNPs and various marine organisms, including primary producers, zooplankton, and higher-trophic-level species, focusing on ingestion, accumulation, translocation, and toxicological effects; (3) emphasize the impacts of MNPs on metabolism, immune function, reproduction, and genetic stability and evaluate associated ecological risks based on the latest research findings; and (4) further explore the synergistic toxic effects of MNPs with co-occurring pollutants, as well as their transfer pathways within marine food webs. The overarching goal of this review is to provide a scientific basis and research guidance for developing a robust ecological risk assessment framework and formulating targeted mitigation strategies.

## 2. Materials and Methods

This review provides a systematic synthesis of the current research on the sources, enrichment, transport and transformation, degradation, and environmental fate of MNPs in marine environments, with the aim of identifying the state of knowledge and remaining research gaps. To ensure transparency of the analytical process, the overall procedures for literature identification, screening, and inclusion followed the PRISMA-ScR reporting guidelines. Literature searches were conducted in Web of Science, PubMed, and ScienceDirect, covering the period from database inception to 1 March 2025, and were restricted to publications in English. The search strategy was built around the core terms “microplastic(s)” and “nanoplastic(s)” and their abbreviations (MPs, NPs), combined with keywords related to the processes and settings addressed in this review, including sources and emissions, enrichment and transport, transformation and degradation, environmental fate, and ecological effects, as well as marine-specific qualifiers such as marine, ocean, coastal, and estuary.

Studies were eligible for inclusion if they focused on MPs, NPs, or MNPs and were conducted in marine-related environmental matrices or had clear relevance for marine extrapolation (e.g., coastal zones, estuaries, open oceans, deep sea, marine sediments, marine organisms, or coastal atmospheric deposition). Eligible studies were required to address at least one of the following themes: sources/emissions, enrichment, transport, transformation or degradation, fate, or ecological effects. To enhance environmental realism, marine field observations/monitoring and mesocosm studies were prioritized when synthesizing evidence on enrichment pathways and ecological effects, whereas laboratory studies were primarily used to elucidate mechanisms and causal pathways and to discuss their inherent limitations. To reduce ambiguity arising from differences in experimental materials across studies, we adopt the following terminology in this review: Model nanoplastics (model NPs) refer to commercially manufactured polymeric nanoparticles with relatively narrow size distributions and regular morphologies, which are commonly used for tracing and mechanistic investigations (typically monodispersed spherical particles). In contrast, environmentally relevant nanoplastics (environmentally relevant NPs) denote nanoplastics that are present in environmental samples or generated through fragmentation and aging pathways that are more representative of environmental processes. These particles are more likely to exhibit irregular morphologies, broad and continuous size distributions, and surface conditioning in natural matrices, including weathering and interactions with dissolved organic matter and/or biofilms. Excluded studies mainly comprised purely terrestrial or freshwater investigations lacking marine relevance, studies focusing solely on macroplastics or solid-waste management without addressing MNPs, conference abstracts and other non-peer-reviewed materials, and records that were irrelevant to the scope of this review or lacked extractable key information.

The initial search yielded 5282 records, of which 214 studies were ultimately retained after screening for comprehensive synthesis. From each included study, information was extracted on study region and environmental matrix, particle types and key characteristics, source and emission pathways, enrichment and transport processes, transformation and degradation mechanisms, and ecological or biological effect endpoints. The evidence was synthesized narratively and organized within an integrated framework encompassing “sources–enrichment–transport and transformation–degradation–fate/effects.” Throughout the main text, environmental observational evidence is clearly distinguished from laboratory-based evidence, and conclusions are framed in accordance with the strength of the underlying evidence. To provide an overview of thematic distributions, a co-occurrence analysis of titles and keywords from the included literature was performed using VOSviewer^®^ (version 1.6.20) to construct a keyword co-occurrence network (Figure S1). These visualizations are used solely for descriptive mapping of research themes and hotspots and not for assessing study quality or inferring causality.

## 3. Sources and Accumulation of MNPs in the Marine Environment

### 3.1. Sources and Accumulation

MNP pollution poses a significant global threat to aquaculture and marine ecosystems due to its wide-ranging ecological impacts. MNPs in marine environments originate primarily from two sources: domestic waste (e.g., packaging, textiles) and industrial activities (e.g., sludge, abrasives, leaks) [30,31]. Over 80% of MNPs derive from plastic bottles, bags, and tire wear, with notable contributions from the textile industry, food packaging, and wastewater treatment plants. Although some facilities can remove up to 95% of MPs, nanoparticles (<10 μm) may still be released into the ocean [32]. Recent studies suggest that plastic recycling/reprocessing facilities may represent an underestimated but important point source of MNPs [33]. The steps of mechanical recycling (e.g., sorting, shredding/grinding, washing, frictional abrasion, drying, and conveying) can generate plastic fragments, fibers, and dust that may enter the environment via multiple pathways: (i) recycling wash water/effluent carrying high MP loads to receiving waters [34], (ii) on-site runoff and particle escape affecting nearby sediments and biota [35], and (iii) airborne emissions of MPs and NPs during operations such as shredding [36]. For example, a pilot assessment of a mixed-plastics recycling facility reported MP abundances in the order of 10^6^–10^8^ per m^3^ in raw recycling wash water, indicating potentially substantial releases, even when on-site filtration is applied [33]. In addition, recent work characterizing emissions during shredding in mechanical recycling has shown the generation and release of airborne MPs and NPs, underscoring the relevance of recycling facilities as combined water–air point sources [36]. These findings support the inclusion of recycling facilities in source inventories and highlight the need for targeted mitigation (e.g., advanced wash water filtration and effective dust capture). Aquaculture is an emerging contributor to MNP pollution. Commonly used plastic-based gear—such as nets, coatings, and protective clothing—can degrade under ultraviolet radiation, temperature changes, biofilm colonization, and mechanical abrasion [37]. This degradation leads to micro- and nanoscale fragments via photo-oxidative reactions that generate oxygen-containing functional groups [38]. In reaction systems that re subjected to continuous mechanical stirring and shear, with UV-irradiated and non-irradiated controls, polymer fragments that have undergone marine environmental weathering can release up to approximately 10^6^ micrometer-scale particles per milligram (expressed as particle number, with size distributions predominantly being in the ~1–100 μm range) [39]. This exceptionally high particle yield primarily arises from the exfoliation and fragmentation of surface cracks and irregular structures on aged plastics under shear stress, with the resulting debris being dominated by low-micrometer-sized particles, thereby markedly amplifying particle numbers on a mass-normalized basis. Personal protective equipment in aquaculture, if mismanaged, can also contribute to MNP inputs [40].

Plastic debris generated by terrestrial activities is widely acknowledged as the predominant source of marine microplastic pollution. It is estimated that approximately 80% of marine MNPs originate from land-based sources, with 70% to 90% ultimately accumulating in marine sediments [41]. Acting as critical hydrological linkages between terrestrial and marine environments, rivers serve as the primary pathways for MNP transport [42], accounting for an estimated 70% to 80% of land-to-sea fluxes [43]. Recent modeling efforts suggest that rivers discharge approximately 500,000 metric tons of plastic waste into the ocean annually [44]. It should be noted that estimates of riverine plastic fluxes to the ocean are largely model-based and are sensitive to assumptions regarding solid-waste statistics, leakage rates, parameterization of river retention and resuspension processes, and system boundaries, such as whether MPs are included [44]. As a result, reported global annual fluxes can differ by several-fold across studies [45]. The magnitude and characteristics of riverine plastic inputs vary substantially across regions. Roughly 80% of the global population inhabits river basins characterized by high macroplastic outputs, largely due to inadequate waste management systems. In contrast, in about 40% of river basins across industrialized regions—including Europe, North America, and Oceania—wastewater discharges represent the dominant source, with microplastics being the prevailing form of plastic pollution [44]. Collectively, these findings highlight the central role of rivers as major vectors of plastic transport at the land–sea interface, underscoring the need for region-specific intervention and global mitigation strategies.

In aquatic systems, MNPs interact with microbes and pollutants to form dense aggregates that gradually settle, forming stable sedimentary layers [46,47]. Sediments thus act as long-term sinks and exposure media for benthic fauna, with strong correlations being observed between MP levels in sediments and benthic organisms [41]. Compared to MPs, NPs pose greater risks due to their higher tissue penetration potential [48]. Moreover, biofilms on MNPs facilitate microbial colonization and alter MNPs’ properties, intensifying ecological stress [49].

Widespread MNP bioaccumulation has been reported across marine taxa. MNPs have been detected in deep-sea fish [50] and zooplankton, including shrimp, copepods, and larvae [51], suggesting ingestion-based food web transfer. Tissue distribution varies by particle size and type: 250 nm polystyrene (PS) MPs accumulate in scallop intestines, while 24 nm PS-NPs spread systemically [52]. In sea urchins, larger particles localize in the esophagus, whereas smaller ones reach internal canals [53]. These patterns reflect species-specific exposure pathways influenced by MNP size, polymer type, and feeding behavior.

### 3.2. Factors Influencing Accumulation

Upon entering marine environments, MNPs exhibit a high surface area and sorptive capacity, enabling adsorption of toxicants [54] and microbial colonization [55]. These processes increase particle density, promoting vertical sinking [56]. Marine sediments serve as the major sink for MNPs [57], especially in surface layers, where bioturbation and sediment porosity drive gradual downward migration [58,59]. Despite plastics’ low intrinsic density, MNPs can accumulate in both surface zones and deep seabeds due to aggregation, hydrodynamics, and long-term physicochemical alteration. Here, long-term physicochemical alteration refers to environmental aging processes (e.g., oxidation/weathering, biofouling/eco-corona formation, and aggregation with organic/mineral particles) that progressively modify particle surface chemistry and effective density/buoyancy, thereby favoring sinking and sediment retention [60]. Deep-sea deposition is now recognized as a widespread phenomenon, contributing to persistent “plastic stratigraphy” in sediments. Species-specific traits affect MNPs’ accumulation routes. In addition to ingestion, surface adhesion is common [61]. While motile or burrowing benthic species may shed particles via movement or sediment friction, sessile organisms retain more adhered MNPs.

Ocean currents and wind are key drivers of MNP dispersal. Subtropical gyres, with stable hydrodynamics, entrap debris over long periods, forming accumulation zones such as the Great Pacific Garbage Patch [62]. On smaller scales, monsoons, tidal currents, and coastal hydrodynamics shape nearshore distribution [63], while wind-driven mixing enhances spatial heterogeneity. Anthropogenic inputs further intensify MNP contamination. Rivers are major conduits for land-based plastics from sewage, runoff, and industrial waste [45]. Direct littering from ports, tourism, and fisheries also contributes, creating regional pollution hotspots. Together, natural processes and human activities determine the dispersal and long-term fate of MNPs.

## 4. Transport Pathways and Environmental Processes of MNPs

**Figure 1:**
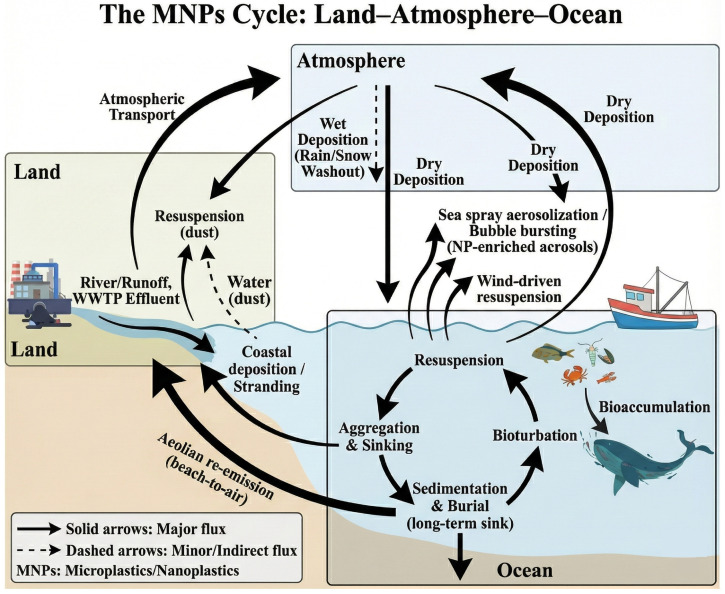
Primary sources and transport transformation mechanisms of MNPs in the marine environment.

MNPs exhibit complex behaviors in marine ecosystems, shaped by physical, biological, and chemical processes (Figure 1). Once in the ocean, they undergo dynamic vertical and horizontal transport influenced by hydrodynamics, atmospheric deposition, biouptake, excretion, and chemical interactions such as aggregation and pollutant adsorption. Sedimentation, bioturbation, and resuspension further modulate their spatial and temporal distribution. Understanding these interlinked processes is critical to assessing MNP-related ecological risks and addressing the ongoing issue of plastic losses from surface waters. Unlike many existing reviews that treat transport and transformation processes separately, this section adopts a source-to-sink perspective to synthesize and couple physical transport, biologically mediated vertical export (the “plastic pump”), and aggregation- and aging-driven changes in effective density and interfacial properties. By explicitly articulating the linkages among these mechanisms, we highlight how their interplay can jointly contribute to the apparent “missing plastics” phenomenon in surface waters and shape the long-term retention of microplastics in benthic environments.

### 4.1. Physical Transport Mechanisms

MNPs undergo significant vertical migration in marine ecosystems, driven by hydrodynamics (e.g., tides, waves, currents) and their intrinsic properties, such as density, shape, and size [64]. Low-density particles generally remain in surface waters but may settle into benthic zones under low-energy conditions, where weak turbulence and limited resuspension allow biofouling- or aggregation-driven increases in effective density to translate into net downward transport and near-bottom retention, while high-density MNPs suspend in midwater before depositing on the seafloor [65,66]. Materials like Polyvinyl chloride (PVC), with negative buoyancy, readily accumulate in sediments. Consequently, MNPs are bioavailable throughout the water column. Although buoyant MNPs can undergo long-range surface transport, their dispersal is influenced by wind and current exposure. Films, fibers, and foams, with high surface-area-to-volume ratios, tend to adsorb pollutants and biofilms, increasing their density and accelerating sinking [67]. In coastal zones, tidal and wave forces modulate MNPs’ distribution, causing shoreline accumulation and re-entry into the sea during tidal cycles [68].

Recent studies highlight the atmosphere as a critical MNP reservoir and transport medium. Airborne MNPs have been detected in both urban and remote regions, confirming their capacity for long-range dispersal and deposition into aquatic systems [69]. Atmospheric transport facilitates cross-ecosystem cycling of plastics and complements dominant riverine and coastal sources [70]. MNPs travel via wind erosion, diffusion, and dry/wet deposition [71], with some studies reporting dispersal distances exceeding 100 km [72]. Environmental factors such as wind direction, particle size, precipitation, and anthropogenic intensity influence airborne MNPs’ distribution [73]. Shifts in wind patterns have been linked to increased local concentrations [74]. For example, in Hamburg, MNP levels rose notably when winds shifted from west to south [75]. Smaller particles (<25 μm) dominate atmospheric MNP profiles due to their higher transport potential [76].

### 4.2. Biological Transport Mechanisms

As MNPs migrate from surface waters to deeper zones and accumulate in coastal sediments, they become increasingly available to particle-feeding organisms [77]. Their small size and resemblance to natural food particles make them easily ingested by marine fauna [78], facilitating trophic transfer through marine food webs. MPs have been detected in a wide range of species, from clams and crabs to fish and whales [79,80]. Besides direct ingestion, MNPs can be transferred indirectly via prey consumption [81], potentially entering the human food chain through seafood [82]. These processes highlight ecological and health concerns related to bioaccumulation and trophic transfer. Studies show that MNP concentrations increase with trophic level, with apex predators exhibiting the highest loads [83,84]. Moore et al. [85] estimated the potential annual intake of microplastic particles by beluga whales via trophic transfer based on measured microplastic abundances in the gastrointestinal tracts of prey fish and the energetic feeding requirements of belugas, yielding an estimated range of approximately 3.8 × 10^3^–1.45 × 10^5^ particles per year. It should be noted that this study did not directly assess the trans-organ distribution of microplastics within beluga whales, nor did it provide empirical evidence for associated toxicological effects. Predators generally show higher loads than filter or deposit feeders, but exceptions exist [86]. The retention time, ingestion frequency, and metabolic rate further modulate accumulation levels [87].

Marine organisms also enhance the downward transport of MNPs by ingesting and egesting them as feces or pseudofeces, thus accelerating vertical flux compared to passive sinking via marine snow or biofilm aggregation [88]. Bivalves and ascidians promote this flux through filtration and biodeposition [89]. Additional contributors include aging fishing gear, planktonic crustaceans, and appendicularians that package particles into fecal pellets or mucus structures [90]. Sediment trap studies confirm enhanced MNP deposition by organisms like *Mytilus edulis*, while benthic bioturbation facilitates burial into deeper sediments [91].

In summary, feeding, egestion, and bioturbation form a biologically driven “plastic pump” that governs the vertical redistribution of MNPs [92]. This mechanism helps explain the “missing plastics” paradox in surface waters and provides a framework for understanding the fate and risks of MNPs. However, the underlying processes remain underexplored, requiring further mechanistic investigation [93].

### 4.3. Chemical Interactions and Aggregation

The density and physicochemical properties of MNPs critically influence their environmental behavior. Heavier particles settle faster, increasing exposure to benthic organisms [93]. Biofilm formation further raises the particle density and promotes vertical sinking. Beyond being pollutants, MNPs serve as vectors for hazardous substances such as heavy metals and POPs due to their high surface area, lipophilicity, and surface roughness [94,95]. Non-polar plastics like polyethylene (PE) and polypropylene (PP) have strong affinities for hydrophobic pollutants such as Polychlorinated biphenyls (PCBs), Dichlorodiphenyltrichloroethane (DDT), and Polycyclic Aromatic Hydrocarbons (PAHs). Environmental aging enhances adsorption of polar substances, including antibiotics and metal ions, through the formation of polar surface groups [96]. The sorption capacity also varies by particle shape; PS microspheres show enhanced metal ion binding, especially in acidic conditions [97].

Aquatic environmental factors modulate MNP adsorption. High salinity compresses the electrical double layer, increasing hydrophobic contaminant binding [98]. pH alters MNPs’ surface charge, affecting interactions with anionic or cationic species [99]. Dissolved organic matter (DOM) competes for binding sites or changes pollutant mobility [100]. These pollutant-laden MNPs can disperse widely, enter marine food webs, and reach humans through seafood consumption [101]. Zooplankton ingestion initiates upward transfer through fish, seabirds, and mammals. Adsorbed toxicants may desorb in host tissues, triggering oxidative stress, apoptosis, and immune disruption [102]. These complexes also persist in sediments, where redox shifts or bioturbation may release pollutants, causing secondary pollution.

### 4.4. Sedimentation and Resuspension

A key unresolved issue is whether MPs deposited onto sediments via feces or pseudofeces can be remobilized after environmental disturbances. Bioturbation typically occurs in the upper sediment layers and varies across species [64]. Many benthic organisms promote further burial of MPs through activities like burrowing, facilitating their vertical transport into deeper strata [103]. This burial reduces the potential for resuspension and leads to MP accumulation in benthic zones, with possible long-term effects on ecosystem structure and function [104]. Moreover, MPs may disrupt sedimentary carbon and nitrogen cycling, weakening the ocean’s carbon sequestration capacity [105].

Despite this, physical disturbances can reverse burial. Storms, wave action, and internal tides can resuspend buried MPs by disrupting surface sediments [106]. In shelf and coastal zones, seasonal currents and extreme weather events increase the resuspension frequency [107], expanding dispersion and elevating biological exposure risks. Low-density polymers like PE and PP often form aggregates with organic matter, but these can disintegrate under turbulence, releasing MPs back into the water column [108]. The sediment type also influences resuspension: clay and silt offer greater retention, while sandy substrates are more prone to particle release [109]. Thus, MP burial is dynamic and reversible, governed by cycles of deposition, disturbance, and redistribution.

### 4.5. Deep-Sea Sedimentation and Final Fate of MNPs

Marine sediments have emerged as major sinks for MNPs, with concentrations significantly exceeding those in surface waters [106,110]. Kane et al. [106] reported up to 1.9 million particles/m^2^ in deep-sea sediments, highlighting the formation of “plastic hotspots”. Zhu et al. [110] estimated that by 2020, 3–11 million metric tons of plastics had accumulated on the seafloor—hundreds of times greater than surface levels (this estimate is primarily based on a limited set of deep-sea observations and relies on extrapolation using statistical or spatial models; the reported range of 3–11 Mt partly reflects uncertainties arising from sparse sampling coverage and pronounced variability in seafloor topography and sedimentary processes in deep-sea environments). This accumulation is driven by physical processes such as thermohaline circulation, ocean currents, and turbidity flows, which facilitate long-range transport and burial of plastics in deep-sea canyons [111]. Additionally, biofilm formation and aggregation increase the density of low-buoyancy polymers like polyester and polypropylene, enhancing their sedimentation [112].

The biological pump also contributes to MNPs’ vertical transfer. Microplastics are incorporated into sinking marine snow, fecal pellets, and other organic aggregates, promoting downward flux. Galgani et al. [92] found microplastics embedded in rapidly sinking particles, accounting for up to 3.8% of particulate organic carbon flux. However, MNPs can disrupt this process. Microfibers reduce the size and sinking velocity of marine snow by up to 50% [112], and copepod fecal pellets containing microplastics show markedly lower carbon export efficiency. Thus, while MNPs facilitate their descent, they may impair the ocean’s carbon sequestration capacity.

Although studies on nanoplastics’ fate in the deep sea remain limited, recent work has confirmed their presence at depths of 300 m [113]. Their small size increases the likelihood of attachment to suspended matter or ingestion by plankton, possibly accelerating their vertical transport. Both sedimentation and biological processes likely govern their redistribution, but further empirical research is needed.

## 5. Degradation and Transformation Processes of MNPs

MNPs, as persistent particles that are resistant to natural degradation, are widely distributed in the environment, underscoring the urgent need to improve plastic waste management systems. A central question is whether the overall fate of microplastics in marine environments is dominated by long-term persistence and accumulation or by degradation and removal. Available evidence generally indicates that, under typical marine conditions, commonly used synthetic polymers remain largely environmentally persistent [114,115]. In most cases, what is described as “degradation” does not imply complete mineralization, but rather reflects weathering- and fragmentation-driven size reduction, accompanied by changes in surface chemistry and bioavailability, rather than rapid elimination at the ecosystem scale [116,117]. Accordingly, the environmental fate of microplastics is more appropriately understood as involving physical and chemical transformation during cross-media transport and gradual accumulation, particularly toward sink compartments such as sediments and the deep sea. It should be emphasized that these processes are strongly environment-dependent: in surface waters characterized by high irradiance and energetic conditions, photochemical reactions and mechanical fragmentation may proceed relatively faster [39,118], whereas under low-temperature, deep-sea, or hypoxic conditions, degradation rates are markedly reduced, allowing microplastics to persist for extended periods within sediments [119,120].

**Figure 2:**
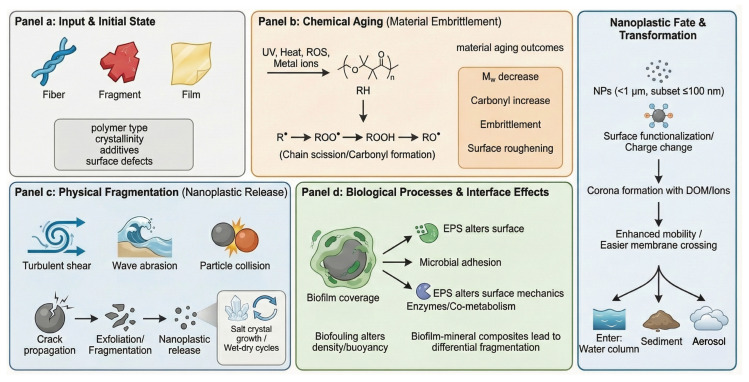
Conceptual schematic illustrating the coupled physical, chemical, and biological processes involved in the transformation of microplastics to nanoplastics in marine environments.

Given that the complete removal of plastics that are already present in the environment is impractical, the current research increasingly focuses on enhancing degradation efficiency through environmentally driven processes or post-collection treatment strategies. To mitigate the ecological and human health risks associated with MNPs, a range of degradation approaches—including photocatalytic, biological, physical, and chemical methods—have been explored, offering potential sustainable solutions to address the long-term environmental impacts of MNP pollution (Figure 2).

**Figure 3:**
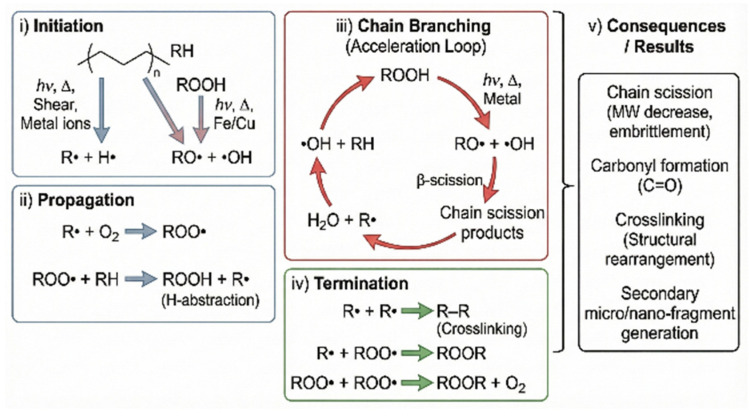
Free-radical chain reactions involved in the auto-oxidation of plastics.

MNPs, as persistent particles that are resistant to natural degradation, can persist in the environment for long periods while undergoing continuous physical and chemical transformations [121]. Under natural conditions, plastics are not entirely inert; their polymer backbones can undergo auto-oxidative reactions when exposed to ultraviolet radiation, heat, mechanical shear, and metal ions [122]. This process is governed by free-radical chain reactions, encompassing initiation, propagation, chain branching, and termination stages, and ultimately leads to molecular weight reduction, structural embrittlement, and the generation of secondary MNP fragments [123] (Figure 3). Understanding these environmentally driven degradation mechanisms provides an essential mechanistic basis for the subsequent development and optimization of photocatalytic, biological, physical, and chemical strategies for MNP degradation and management [124].

### 5.1. Physical Degradation

UV photodegradation is a major driver of plastic aging and fragmentation. UV radiation promotes the formation of oxygen-containing groups (e.g., ketones, carboxylates, hydroxyls), reducing hydrophobicity and initiating surface cracking, discoloration, and brittleness [125]. PS and PE are particularly sensitive, forming free radicals that trigger chain scission and particle breakdown [126]. However, UV penetration is limited to surface microlayers, often requiring other physical processes to accelerate degradation.

Mechanical abrasion, especially in coastal zones, contributes significantly to particle size reduction. Wave action, sand collision, and anthropogenic activities (e.g., fishing, shipping) erode particle edges and enhance fragmentation into nanoplastics [127]. This process increases surface roughness, facilitating pollutant adsorption and microbial colonization. A single gram of synthetic fiber can release tens to hundreds of thousands of microfibers through abrasion.

Thermal stress also accelerates degradation, particularly in high-temperature or tropical waters. Heat weakens polymer chains, enhances thermal oxidation, and lowers material stability, especially in plastics like PS and PVC [128,129]. PE is more thermally sensitive than polyethylene glycol terephthalate (PET), which is comparatively stable.

Physical fragmentation facilitates other degradation mechanisms by increasing the surface area. Natural shear forces from waves and wind can reduce particle sizes, especially in estuarine and beach environments [130]. Interactions with sand and shells and freeze–thaw cycles promote surface cracking and molecular cleavage [131].

Degradation in nature is driven by coupled processes. UV-induced cracks are exacerbated by mechanical and thermal forces, accelerating fragmentation and reactivity [132]. As the particle size declines, the specific surface area increases, enhancing mobility, bioavailability, and pollutant adsorption and thereby raising ecological risks.

Coagulation–flocculation offers a physical removal strategy by inducing particle aggregation and sedimentation. Although the raw MNP removal efficiency is low (5–10%) [133], it improves with pretreatments like UV exposure or microbial conditioning that enhance surface anchoring sites [134].

### 5.2. Chemical Degradation

Chemical degradation is a key transformation pathway for MNPs, involving chain scission or crosslinking induced by chemical agents such as peroxides or carbonyl compounds, which reduce molecular weight and stability [135]. Advanced oxidation processes (AOPs), including treatments with ozone, Fenton’s reagent, and activated persulfate, generate reactive oxygen species that oxidize surface functional groups on plastics like PS and LDPE, promoting degradation [136].

Oxidation introduces polar groups (e.g., hydroxyls, carbonyls), enhancing biodegradability and pollutant adsorption [91]. It proceeds via free radical chain reactions through initiation, propagation, branching, and termination stages and is influenced by oxygen concentration, UV exposure, temperature, and polymer properties [137]. Non-polar polymers like PE and PP are especially susceptible to oxidative aging.

Hydrolysis occurs in polymers with hydrolyzable bonds, such as esters and amides, which are found in PET, polylactic acid (PLA), and polyurethanes. It involves nucleophilic attack by water, cleaving polymer chains into oligomers and enhancing biodegradation [138]. Hydrolysis is affected by pH, temperature, and catalysts such as metal ions and organic acids, with alkaline conditions favoring ester bond cleavage [139].

Photodegradation dominates at the ocean surface, where UV-A and UV-B break C-C and C-H bonds, generating free radicals that lead to yellowing, cracking, and fragmentation [126]. Its efficiency depends on light intensity, polymer type, color, and additives [123].

Photocatalytic degradation utilizes catalysts (e.g., TiO_2_, ZnO) under light to generate reactive oxygen species, enabling deep oxidation. ZnO nanorods and light-driven nanomotors have shown high PS degradation efficiency within days [140]. UV pretreatment improves photocatalytic reactivity by enhancing surface activation [141].

Thermal degradation and pyrolysis break down polymer chains via heat, with pyrolysis being conducted in inert atmospheres at 300–900 °C and producing small molecules or fuels [142]. Thermal degradation alters polymers’ structure, crystallinity, and mechanical properties, often serving as pretreatment before biodegradation [105]. Applications include Pyr-GC/MS for MP analysis, syngas generation, and enhancing degradation under low-temperature marine conditions [143]. Thermal treatment at 800 °C has achieved high carbon conversion rates, especially with metal salts present [144].

Chemical degradation transforms plastics through a sequence of surface oxidation, chain scission, fragmentation, and low-molecular-weight release, altering key environmental behaviors, including density, charge, and bioavailability.

### 5.3. Biological Degradation

Plastic biodegradation refers to the process by which microorganisms, such as bacteria and fungi, form biofilms on the plastic surface. They secrete specific enzymes that break down the polymer backbone and side chains. This leads to the depolymerization of plastics into oligomers and dimers. It can ultimately generate products such as carbon dioxide and water. If the degradation is not fully mineralized, it may result in organic or inorganic metabolic or transformation products. The nature of the degradation products depends on environmental conditions. Under aerobic conditions, the primary products are CO_2_ and H_2_O. Under anaerobic conditions, methane and CO_2_ are produced. The biodegradability of plastics is influenced by several factors. These include chemical structure, molecular weight, surface roughness, hydrophilicity/hydrophobicity, hardness, and morphology. Even low-molecular-weight MPs can become more biodegradable after artificial weathering. Heat or UV exposure can enhance pre-oxidation activity, making them more susceptible to biodegradation. Current research focuses on multiple biodegradation pathways. These involve bacteria, fungi, enzymes, combined microbial systems, and algae. Each pathway exhibits unique characteristics in degradation efficiency and adaptability.

#### 5.3.1. Bacterial Degradation

Bacteria from diverse environments have been demonstrated to degrade MNPs to the level of mineralization [145]. This capability has particularly attracted attention in marine environments, where bacterial attachment to MNPs and their degradation potential are significant areas of focus (Table S1). Lv et al. [146] discovered that Alcanivorax xenomutans and Halomonas titanicae effectively degraded PS, while continuously releasing considerable quantities of MNPs, with particle concentrations reaching as high as 7.64 × 10^6^ particles/L. These two strains, respectively, converted 1.3% and 1.9% of PS into MNPs but only achieved mineralization rates of 4.5% and 1.9%. These findings suggest that although microbial degradation can reduce plastic waste, it also contributes to the release of MNPs, thus posing a significant secondary pollution threat. In estuarine environments, Bacillus and Pseudomonas are the dominant bacterial genera in MNP-attached microbial communities [147], which aligns with previous studies indicating that these genera exhibit strong plastic degradation potential [148]. Similarly, various studies have identified bacterial communities on MPs in rivers, highlighting the presence of potential plastic-degrading microorganisms [149]. Furthermore, some degradation processes are not directly carried out by bacteria but are closely linked to the host’s microbiota. For example, Bacillus strains isolated from Zophobas morio larvae can effectively degrade polystyrene [150], while symbiotic bacteria in the citrus mealybug degrade polyethylene [151]. However, individual bacterial strains typically degrade only one or a few types of plastics, and their degradation efficiency is considerably lower compared to mixed microbial communities, particularly when degrading complex mixtures of MPs. Given the intricate composition of MPs and their prior environmental degradation, the capability of single bacterial strains to degrade them remains significantly limited.

#### 5.3.2. Fungal Degradation

Fungi, as a crucial microbial group, demonstrate significant potential in plastic biodegradation (Table S2). Research has shown that fungi can metabolize various synthetic polymers, including PP and PET, using them as their sole carbon and energy sources [145]. Among these, Ascomycota are the most active [145]. In experimental studies, the marine fungus Zalerion maritimum was able to grow on PE as a substrate, leading to a significant reduction in both particle mass and size [152]. Similarly, the filamentous fungus Fusarium oxysporum was found to degrade polyester fibers in inorganic media and exhibited some erosive effects on the plastic substrate [153]. The degradation abilities of fungi are not only attributed to the non-specific enzymes that they secrete, which break polymer chains, but are also closely related to their unique attachment and penetration abilities. Hydrophobic proteins secreted by fungi promote the attachment of fungal hyphae to hydrophobic plastic surfaces, and these hyphae can penetrate and decompose the three-dimensional matrix of plastics. Unlike bacteria that rely on specific single enzymes, fungi can demonstrate enhanced degradation efficiency and environmental adaptability through the synergistic action of complex multi-enzyme systems.

#### 5.3.3. Enzymatic Degradation

Enzymatic degradation is one of the key mechanisms by which microorganisms break down plastics (Table S3). Nearly all polymer degradation processes require enzyme involvement [154]. While enzymatic degradation reactions generally take longer and depend on specific catalytic conditions, their high selectivity and environmental friendliness make them a vital tool for addressing MNP pollution [155]. The enzymes that are primarily involved are hydrolytic enzymes, which act on hydrolyzable chemical bonds, such as ester and amide bonds, within polymers [146]. In unique ecosystems like mangroves, fungi secrete extracellular enzymes, including laccases, manganese peroxidases, and lignin peroxidases, which promote the degradation of PE films [156].

Different plastic types involve distinct enzymatic systems for degradation. For instance, PE degradation depends on laccases and peroxidases; PVC degradation relies on lignin peroxidases; PS degradation involves esterases and non-heme peroxidases; and PET degradation has identified key enzymes such as carboxylesterases and lipases [157]. These degradations typically result from the synergy between extracellular and intracellular enzymes, with extracellular enzymes initiating the hydrolysis process by modifying the hydrophilicity of the plastic surface, a crucial step in the initial breakdown of MNPs [158]. However, natural enzymes face challenges in terms of catalytic efficiency and stability. Enzyme engineering techniques, such as directed evolution and structural optimization, are widely used to improve their performance [159]. Although engineered enzymes still need further enhancement in catalytic activity, developments in deep learning and synthetic biology are accelerating their practical application in plastic pollution remediation [160].

#### 5.3.4. Microbial Synergistic Degradation

Although individual microbial strains can degrade specific types of plastics, their overall degradation efficiency is often limited, and certain degradation byproducts may exhibit toxicity to the microbes themselves, thereby constraining their long-term degradation potential [161]. To address these challenges, co-degradation strategies utilizing multi-strain microbial consortia have been developed. These consortia offer several advantages: (1) intermediate degradation products can be metabolized by other strains, minimizing the accumulation of toxic compounds [162]; (2) the metabolic diversity of the consortium reduces substrate specificity constraints, allowing for the degradation of a broader range of plastic types; and (3) synergistic interactions among microbial members enhance the degradation efficiency and improve environmental resilience.

A variety of co-degradation systems have been developed to date. For instance, Taniguchi et al. [153] proposed three distinct strategies for the biodegradation of PET, while Park et al. [163] isolated a mixed microbial consortium composed of Bacillus and Corynebacterium species from a landfill, which effectively accelerated the degradation of PE-MPs. Although such co-degradation systems have demonstrated considerable success in enhancing degradation efficiency, they involve complex interactions among multiple microbial species and enzymatic pathways, which remain challenging to regulate. Therefore, a deeper understanding of the key influencing factors and underlying mechanisms is urgently needed to optimize and effectively apply these systems.

#### 5.3.5. Algal Degradation

Algae, which include both microalgae and macroalgae, are photosynthetic autotrophs that are capable of adapting and thriving under a wide range of environmental conditions. In recent years, they have emerged as promising biological agents for the remediation of MNP pollution [164]. Due to their rapid growth rates, low cultivation costs, high stress tolerance, and elevated biomass productivity, algae have been effectively employed in the removal of various environmental contaminants, including heavy metals, pesticides, pharmaceuticals, and plastic particles [165].

Algae participate in the degradation of plastics through various mechanisms, including physical adsorption, bioaggregation, enzymatic action, and synergistic interactions with coexisting microorganisms. Their cell surfaces are enriched with negatively charged polysaccharides, which facilitate strong adsorption of positively charged plastic particles [166]. The branched morphology of algal cells also enables them to function as natural “biofilters” [167]. Certain macroalgae enhance plastic aggregation and sedimentation by secreting extracellular polymeric substances [168]. The diatom Navicula pupula and the green alga Chlamydomonas reinhardtii have been observed to colonize plastic surfaces and form biofilms that promote degradation [169]. Additionally, some green algae secrete non-specific enzymes such as lipases and esterases, which can cleave polymer ester bonds, producing low-molecular-weight fragments that can be further degraded by symbiotic bacteria [170]. Research suggests that algae enhance microbial plastic degradation by secreting organic compounds that create favorable microenvironments, thereby stimulating the activity of cohabiting bacteria and fungi to establish synergistic degradation consortia [171]. For instance, Dunaliella tertiolecta maintains high photosynthetic efficiency under elevated concentrations of MPs [172], while *Microcystis aeruginosa* exhibits significant oxidative stress responses when exposed to positively charged plastic particles [173].

Although algae have demonstrated the ability to degrade a variety of plastic types [168], their degradation efficiency is influenced by environmental factors such as the light intensity, particle concentration, and physicochemical properties of the plastics. NPs, in particular, may penetrate algal cell membranes, triggering the generation of reactive oxygen species (ROS) and inducing cytotoxic effects [166]. To overcome these limitations, recent studies have focused on genetically engineering algae to express plastic-degrading enzymes such as PETase, thereby enhancing their degradation potential [174]. Furthermore, membrane bioreactor (MBR) systems that combine algae with membrane filtration technologies have achieved MP removal efficiencies of up to 99%, offering a promising approach for wastewater treatment and environmental remediation [175]. In conclusion, algae exhibit multifunctional roles in the mitigation of MNP pollution through mechanisms such as adsorption, aggregation, enzymatic degradation, and synergistic interactions with microbes. These capabilities position algae as a critical frontier in the development of next-generation technologies for marine pollution remediation.

### 5.4. Combined Degradation

Although progress has been made in degrading plastics through physical, chemical, and microbial methods, the high crystallinity, hydrophobicity, and thermal stability of polymers still limit their degradability in natural environments [125]. For instance, PE has a semi-crystalline structure, with crystallinity between 20% and 40%, which enhances its mechanical strength but lowers microbial susceptibility [176]. In addition, the hydrophobic surface of plastics hinders microbial adhesion and biofilm formation, impeding the initiation of biodegradation [177].

To address these barriers, recent studies have explored combined strategies using physicochemical pretreatment to improve plastic’s surface properties and support microbial activity [125]. UV irradiation is widely applied, as it promotes oxidation, introduces carbonyl and hydroxyl groups, reduces molecular weight, and increases surface hydrophilicity [176]. After UV treatment, microbial consortia were shown to reduce the molecular weights of PS and PE by 5.1% and 7.8%, respectively, over 45 days [125]. Pretreatment also alters plastic’s structure, exposing amorphous regions for preferential microbial degradation, which can increase the residual crystallinity [134]. In a study using UV-aged PP and a nitrifying-denitrifying consortium, carbonyl and hydroxyl indices increased substantially, and nitrogen removal reached 99%, indicating synergistic effects between plastic modification and pollutant removal [134]. In summary, combining pretreatment with microbial degradation holds promise for improving the transformation of persistent plastics in the environment.

### 5.5. The Impact of Environmental Conditions on MNP Degradation

Environmental conditions critically influence the degradation of MNPs, interacting with polymer properties such as crystallinity, hydrophobicity, and surface charge [125]. Temperature governs the polymer chain mobility and reaction rates; elevated temperatures accelerate both chemical and enzymatic degradation, with polyester plastics like PET and PLA showing enhanced breakdown between 15 °C and 35 °C [141]. In tropical waters, high temperatures favor photochemical oxidation and hydrolysis, but non-polar plastics such as PE and PP often require additional UV radiation or oxidizing agents for effective degradation [126]. Oxygen availability also shapes degradation pathways. Aerobic conditions promote oxidative cleavage and microbial enzyme activity, while deep-sea or anoxic zones reduce microbial diversity and limit degradation to about one-third of aerobic rates [156]. pH affects degradation through electrostatic interactions. Acidic environments enhance plastic adsorption and oxidation but may inhibit microbial activity [178], whereas alkaline conditions can reduce adsorption and interfere with degradation due to electrostatic repulsion and the presence of humic substances [179]. Light, particularly UV, accelerates surface aging and fragmentation, driving secondary microplastic formation in sunlit areas like beaches and intertidal zones [180]. Visible light can indirectly support degradation by activating photosensitizers [181].

Nutrient concentrations regulate microbial communities and enzyme secretion. While moderate nutrient availability enhances the activity of degraders such as lipase- and esterase-producing microbes [182], excessive nutrients may disturb the microbial balance and reduce plastic’s bioavailability [104]. Notably, environmental variables often interact. For example, a low pH and low temperature may synergistically enhance plastic adsorption and reactivity by increasing the surface area and interaction with elements like carbon and nitrogen [183]. In contrast, cold, deep-sea, or oxygen-deficient waters are characterized by extremely low degradation rates, allowing plastic particles to persist in sediments over long timescales [108]. Understanding these complex interactions is essential for predicting MNPs’ behavior and designing realistic strategies for environmental mitigation.

### 5.6. Mechanisms of MPs’ Transformation into NPs

The transformation of MPs into NPs follows a complex, multi-phase degradation process driven by physical, chemical, and biological forces, with fragmentation as the key transitional step (Figure 3). This begins with surface microcracks caused by ultraviolet radiation, mechanical abrasion, and thermal stress, which expand over time and reduce the particle size [126]. UV-B radiation induces photo-oxidative reactions that generate free radicals, break polymer chains, and increase brittleness, making materials more vulnerable to mechanical disintegration from waves, collisions, and wind [184]. This “outside-in” pattern of degradation, where surface weathering precedes internal breakdown, is well documented in low-density plastics like PE and PP, which begin edge fracturing and particle detachment within months of exposure [127]. Resulting fragments often show a right-skewed size distribution, indicating the generation of large quantities of submicron particles. As the size decreases, the surface area increases, promoting further oxidative, hydrolytic, and microbial degradation [185]. Fragmentation rates vary by polymer type; for instance, PS and PVC, with higher glass transition temperatures and chain rigidity, degrade faster than PE or PP [96]. Additives such as plasticizers and antioxidants may leach or migrate to the surface during degradation, affecting crack propagation and particle size evolution [186].

The formation of nanoscale particles alters the physicochemical behavior of plastics, resulting in intensified Brownian motion, increased bioavailability, enhanced penetration across biological membranes, and elevated reactivity and toxicity in aquatic environments [71]. Laboratory studies confirm that long-term UV exposure and hydrodynamic agitation can release substantial quantities of NPs sized between 1 and 100 nm, consistent with those found in the environment [187]. This transformation process encompasses UV-induced chain scission, mechanical fragmentation, and chemical degradation, all modulated by factors such as crystallinity and glass transition temperature. Environmental conditions like temperature, solar radiation, and turbulence further interact with polymer characteristics to determine degradation rates and size distribution. Understanding these dynamics is crucial for identifying sources of environmental nanoplastics and assessing their ecological risks.

## 6. Ecological and Biological Impacts of MNPs

### 6.1. Bioavailability and Uptake Pathways

#### 6.1.1. Mechanisms of MNP Ingestion by Marine Organisms

**Figure 4:**
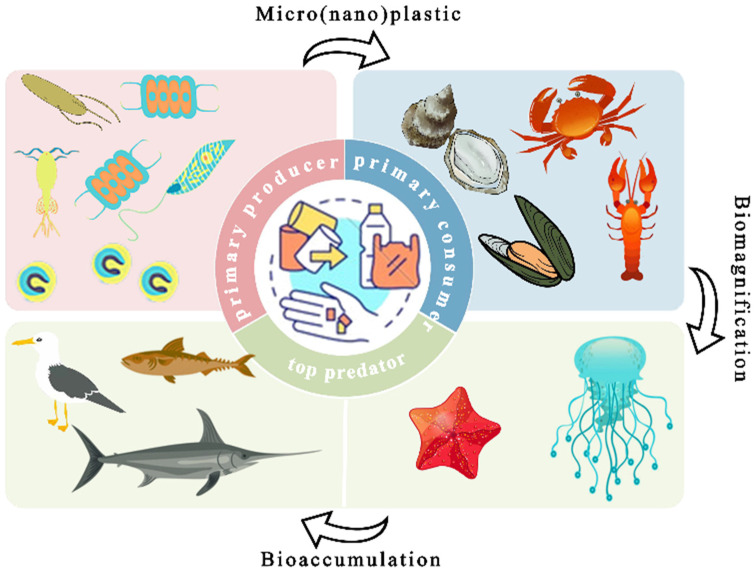
Trophic transfer and pathways of MNPs in marine ecosystems.

MNPs are widespread in marine environments and exhibit high bioavailability due to their broad size range and extensive distribution (Figure 4). Their particle sizes overlap substantially with those of natural food items and suspended particulate matter, making them accessible to a wide range of marine organisms. However, reported microplastic concentrations in the ocean span several orders of magnitude across regions and depth layers (e.g., approximately 10^−4^ to 10^4^ particles m^−3^ in compiled water column datasets [188]) and are generally much lower in the open ocean than in nearshore, estuarine, or convergence “hotspot” environments. Consequently, statements regarding bioavailability and associated effects should be interpreted in a scenario- and dose-dependent manner, with careful distinction between hazards observed under laboratory conditions and ecological risks under environmentally relevant exposure levels.

Bioavailability, defined as the capacity for ingestion, absorption, and physiological integration, is influenced by factors such as particle size, shape, surface chemistry, density, and interactions with organic matter or pollutants [189]. MPs are commonly taken up by marine organisms through non-selective ingestion, filter feeding, mucus entrapment, or accidental consumption. Filter feeders, including zooplankton, bivalves, and fish, have been widely reported to ingest MPs, with many species mistaking them for food particles [81]. In mussels and zooplankton, MPs are captured during filtration and transported to digestive tissues, where they may accumulate and persist for weeks, being partially eliminated through excretion but potentially disrupting immunity and metabolism [190]. Compared to MPs, NPs exhibit enhanced bioavailability due to their small size and high surface reactivity, allowing them to penetrate biological barriers and reach internal tissues and organelles, raising complex toxicological concerns [191]. In fish, MNP uptake can occur via ingestion or gill filtration, as similarities in size, shape, or color between plastics and prey may lead to mistaken ingestion [192]. Stomach content analyses confirm the widespread presence of MNPs across various trophic levels, highlighting the potential for trophic transfer in marine food webs [193]. Furthermore, plastic particles are often coated with a biologically active “eco-corona” composed of microorganisms, algae, and organic matter, which may enhance the likelihood of ingestion by mimicking natural food sources [194].

#### 6.1.2. The Impact of Particle Size and Polymer Type on Ingestion

The uptake of MNPs by marine organisms is strongly influenced by their physicochemical properties, particularly their particle size, polymer type, density, and morphology. These traits govern ingestion likelihood, biological accessibility, and internal distribution. Studies show that bivalves can selectively filter particles based on size and surface features, often rejecting unsuitable MPs as pseudofeces rather than retaining them [195]. Particle size is a key determinant: larger MPs (>100 μm) are typically ingested by mussels, polychaetes, and filter-feeding fish, while nanoscale particles more easily bypass feeding barriers, enter internal tissues, and accumulate. Their high surface-area-to-volume ratio enhances both their chemical reactivity and capacity to adsorb pollutants [11]. NPs may pass through gill structures and translocate to the gastrointestinal tract, potentially inducing tissue damage and histopathological changes [196].

Polymers’ identity also affects their uptake and internal fate. The polarity, lipophilicity, and surface charge of each polymer influence interactions with biological membranes, proteins, and environmental contaminants. For example, lipophilic polymers such as PS and PVC exhibit high affinity for hydrophobic pollutants and readily form eco-coronas, increasing their potential for ingestion [194]. In contrast, polar polymers like PET interact more with proteins and microbial communities, facilitating cellular internalization [102]. Additionally, polymers may release additives or degradation byproducts such as phthalates or bisphenol A (BPA), which can alter organism feeding behavior via olfactory or chemosensory signals [197].

### 6.2. Cellular and Physiological Impacts

#### 6.2.1. Oxidative Stress

MNPs are widely ingested and accumulated by marine organisms, leading to substantial health risks across diverse taxa [198]. Beyond causing physical damage, MNPs induce cellular responses, particularly oxidative stress, a major toxicological pathway [30]. Co-exposure to MNPs and other pollutants, such as pesticides and pharmaceuticals, can disrupt redox balance by increasing reactive oxygen species (ROS) levels beyond the buffering capacity of antioxidant systems, resulting in oxidative damage to lipids, proteins, and DNA [199]. ROS may originate from UV-induced photo-oxidation on plastic surfaces or from pro-oxidant plastic additives migrating to the surface [200]. Once internalized into tissues or bloodstream, MNPs further elevate ROS concentrations [201], overwhelming antioxidant defenses such as superoxide dismutase (SOD), a key enzyme that is responsible for neutralizing ROS and maintaining redox homeostasis [202]. Prolonged exposure to MNPs can suppress SOD activity, impair immune responses, and weaken physiological resilience [203].

Oxidative stress caused by MNPs interferes with metabolism and oxygen regulation, triggering lipid peroxidation and protein carbonylation, which lead to systemic cellular injury [204]. Chronic exposure affects development, reproduction, and immune competence. MNPs can also serve as carriers for pathogenic microbes, exacerbating oxidative stress upon ingestion [205]. Pathogen-associated MNPs activate immune responses, increase ROS production, and contribute to tissue damage. Microfibers often accumulate in the gills, causing irritation, impaired respiration, and combined oxidative and inflammatory responses [206]. Moreover, MNPs readily adsorb heavy metals and POPs, compounding exposure risks [207]. Experimental studies on Pacific oysters demonstrate that exposure to PE and PET MPs induces dose-dependent changes in SOD activity: low doses stimulate adaptive antioxidant responses, while high doses lead to antioxidant exhaustion [208].

#### 6.2.2. Metabolic Disruption and Immune Response

Upon ingestion, MNPs can induce oxidative stress, DNA damage, immune dysfunction, and metabolic disturbances, affecting both gastrointestinal and peripheral systems and often leading to local or systemic inflammation [209]. Studies on polychaetes show intestinal inflammation and suppressed immunity upon MP exposure [210], while systemic circulation of MNPs may trigger hypersensitivity reactions and disrupt immune homeostasis [211]. In teleosts such as *Oryzias latipes* and *Pomatoschistus microps*, MNPs inhibit acetylcholinesterase (AChE) activity, impairing neurotransmission and inducing neurotoxicity and behavioral changes [212]. These effects span the cellular, tissue, and molecular levels, suggesting widespread physiological disruption.

Environmental conditions can significantly influence the toxicity of MNPs. For example, a 14-day co-exposure to polystyrene MPs and low salinity intensified stress responses in the gills of Crassostrea gigas, affecting their metabolism and antioxidant defenses [213]. In *Litopenaeus vannamei*, even low-dose PS-MP exposure altered hepatopancreatic metabolism, disrupting the glycolysis, lipid, and amino acid pathways [214]. MNPs also impair reproduction across species. PS-MPs reduced reproductive output in *Calanus helgolandicus* [215], while phthalate plasticizers are linked to endocrine disruption in aquatic organisms [216]. Seabirds consuming MPs have shown delayed reproduction [217], indicating that MNP-induced metabolic disturbances may lead to long-term ecological consequences across trophic levels.

### 6.3. Impacts of MNPs on the Marine Food Web

MNPs can enter aquatic food webs either through direct environmental uptake or via trophic transfer through predator–prey interactions [218]. The degree of accumulation in predators is linked to feeding strategies [219]. Once ingested, MNPs can accumulate in various tissues, posing health risks. In fish, MPs are often found in gills [220], while smaller NPs can bypass physiological barriers and reach sensitive organs such as the brain and gonads [221]. MPs frequently accumulate in the liver, whereas NPs tend to remain in intestinal tissues, possibly due to adhesion to microvilli or translocation across epithelial barriers following fragmentation [222]. MPs can also damage intestinal structure and spread via circulation [223]. Tissue-specific accumulation is influenced by species traits, polymer type, exposure time, and concentration. Notably, NPs show greater tissue penetration, including the ability to cross the blood–brain and gonadal barriers, reflecting their higher cellular invasiveness [221].

Intrinsic biological traits further shape MNPs’ distribution. Variation in respiratory structures, membrane permeability, and feeding strategies across aquatic species affects their accumulation potential. Organisms with specialized gill or feeding systems, such as *Corbicula fluminea*, show elevated nanoparticle uptake [224]. Filter feeders like *Daphnia* are especially vulnerable to non-selective ingestion due to their inability to distinguish particles [225]. In contrast, predators acquire MNPs through trophic transfer, promoting their movement across food chains [219]. While feeding mechanisms have been studied in relation to MP uptake [226], the pathways of NPs’ tissue translocation and distribution remain insufficiently understood and warrant further investigation.

### 6.4. MNP-Induced Changes in Microbial Communities

Upon entering aquatic environments, MPs are rapidly colonized by diverse microbial communities that form complex biofilms, collectively referred to as the Plastisphere [227]. These biofilms are compositionally distinct from surrounding microbial populations and often enriched with taxa that are specific to particular plastic types [156]. MPs’ strong adsorption capacity enables them to concentrate marine pollutants and promote biofilm formation [228]. Biofilms increase the particle density and alter surface properties, enhancing the likelihood of MPs sinking and being ingested by benthic organisms. They may also affect the hydration and interfacial behavior of nanoplastics, influencing their transport and fate. Critically, Plastisphere microbes contribute to MNP biodegradation. Environmental factors such as light, suspended matter, and organic carbon affect biofilm development and enzymatic activity, promoting degradation [146]. Notable degraders include bacteria like *Alcanivorax borkumensis* and *Ideonella sakaiensis*, with the latter being capable of depolymerizing PET into monomers [169], as well as fungi such as *Zalerion maritimum*, which utilize hydrophobins to bind and transform hydrophobic plastics [145].

Beyond biodegradation, MPs act as vectors for microbial dispersal (Table 1), including pathogenic species [229]. Plastic-associated biofilms often harbor opportunistic pathogens, forming reservoirs that facilitate the spread of infections, antimicrobial resistance, and toxins [230]. These communities can co-migrate with adsorbed contaminants across aquatic systems, threatening ecosystem health. The Plastisphere also promotes horizontal gene transfer, with MPs providing a stable environment for microbial interactions and potential antibiotic resistance gene (ARG) exchange [231]. This genetic exchange may extend beyond aquatic life to human exposure via ingestion, inhalation, or dermal contact [232], representing a complex risk to both environmental and public health.

### 6.5. Field and Mesocosm Evidence in Marine Systems

Field observations and mesocosm studies provide direct evidence that MNPs are widespread across distinct marine compartments and can accumulate in both biota and sediments under near-environmental conditions [233]. In situ datasets further reveal pronounced spatial heterogeneity, with enrichment hotspots frequently occurring in the sea surface microlayer, coastal convergence zones, estuarine regions, and depositional environments such as shelf and deep-sea sediments [234] (Table S4). Importantly, results from field and mesocosm investigations largely corroborate several key fate processes proposed by mechanistic studies. For example, MNPs can aggregate with natural particles and extracellular polymeric substances, and biofouling subsequently increases their effective density, promoting downward transport from surface waters [235]. A fraction of particles may also become incorporated into marine snow and the biological pump, enabling vertical transfer from the surface ocean to the mesopelagic and benthic realms [92]. In addition, physical structure and benthic–pelagic coupling in systems such as seagrass meadows, coral reef frameworks, and sediment–water interfaces can further shape habitat-specific retention and redistribution patterns of MNPs [236]. Collectively, these real-world observations support the pelagic–benthic continuum perspective, indicating that the transport, transformation, and deposition of MNPs do not follow a strictly linear pathway but instead involve dynamic exchanges among compartments that are governed by hydrodynamics, particle properties, and biological mediation [233].

Nevertheless, attributing the ecological effects of MNPs under in situ conditions remains challenging. Environmental exposures are typically chronic and highly heterogeneous and often intertwined with co-occurring stressors such as warming, hypoxia, and acidification, as well as complex natural particle backgrounds that can obscure or mimic MNP-related signals [237]. Methodological variability among studies—particularly in sampling strategies, size fraction coverage (notably for the nanoscale), and polymer and shape characterization—further limits cross-study comparability and weakens dose–response inference [238]. To narrow the gap between laboratory-based mechanistic insights and environmental realism, future research should advance along several key directions. These include (i) establishing standardized detection and reporting frameworks for the nano-fraction and the smallest microplastic size classes; (ii) aligning particle characterization with field observations by systematically accounting for polymer type, size distribution, aging state, surface chemistry, and biofilm coverage; (iii) implementing exposure scenarios that reflect realistic pathways such as ingestion, trophic transfer, and sediment contact; and (iv) developing multi-stressor mesocosm experiments that incorporate natural particles and background contaminants while maintaining tractable causal structure [239]. Such a “field–mesocosm–mechanism” integrative framework will strengthen causal inference at ecosystem scales and facilitate more robust linkage between hazard signals and real-world exposure scenarios [240].

In real marine systems, evidence related to so-called “systemic effects” is derived mainly from field surveys and mesocosm studies, which typically quantify organismal MNP burdens alongside multiple physiological or biochemical response endpoints [241]. Under exposure conditions that are close to those encountered in the environment, several studies have reported consistent associations between internal plastic particle loads and indicators of oxidative stress, immune or inflammatory responses, and alterations in energy metabolism [241]. It should be noted, however, that such evidence is largely correlational and is often difficult to disentangle from the influence of co-occurring stressors, including temperature variation, hypoxia, acidification, and complex natural particle backgrounds. For nanoscale particles in particular, uncertainties associated with in situ detection and quantification remain substantial, and field-based studies therefore provide limited direct support for trans-barrier transfer or fully resolved causal pathways [242]. Accordingly, in this review, field and mesocosm evidence is used primarily to assess environmental realism and the consistency of response patterns across endpoints, whereas statements regarding “systemic effects” are framed cautiously in terms of possible or potential outcomes; mechanistic interpretation and causal inference are discussed mainly on the basis of laboratory studies [243].

### 6.6. Analytical Uncertainty and In Situ Detection Limitations for Nanoplastics

Integrating environmental exposure concentrations (e.g., MEC or PEC) with effect thresholds—such as PNECs derived from NOEC, EC10, or EC50 values, or HC5 values obtained from species sensitivity distributions (SSDs)—is a commonly used framework for scenario-based risk characterization (e.g., RQ = PEC/PNEC) and, in principle, allows for comparisons across environmental compartments and marine settings [240]. However, in the current body of marine MNP research, substantial heterogeneity remains in particle size definitions, characterization approaches, exposure metrics (particle number, mass, or surface area), and the reporting of effect endpoints. In addition, uncertainties associated with the in situ detection and quantification of NPs further constrain the rigorous numerical integration and harmonized quantification of results across studies [244]. Accordingly, in the present review, the risk discussion focuses on outlining the conceptual logic and applicability boundaries of this integration framework, while the systematic integration of PEC and PNEC/SSD data based on standardized datasets and their quantitative comparison across scenarios is identified as a key priority for future research [245].

Measured environmental concentrations of NPs are generally low, their particle sizes are extremely small, and sample matrices are often highly complex. As a result, sampling, pretreatment, and separation or purification steps can readily become sources of uncertainty [242]. In many studies, sample handling itself has made a substantive contribution to overall analytical variability. At the same time, differences between laboratory studies and environmental monitoring in analytical workflows, blank control, and data reporting further reduce comparability across studies, limiting the ability to constrain real-world exposure levels [246]. These issues are closely linked to the methodological heterogeneity and lack of standardization that currently characterize this field and are increasingly recognized as major contributors to uncertainty in ecological risk assessment [239].

At the nanoscale, background contamination represents a particularly significant challenge. Contamination from airborne fibers, laboratory equipment, and reagents is often difficult to fully eliminate, and insufficient blank correction or quality control can readily lead to false positives or overestimation in low-concentration samples [247]. More importantly, there is currently no single analytical technique capable of reliably providing, at the nanoscale, comprehensive information on polymer composition, particle size or morphology, and abundance or concentration simultaneously [248]. In practice, multiple complementary techniques are therefore required; however, differences among these methods in detection limits, selectivity, and quantitative capability substantially increase the complexity of data interpretation [248].

Against this background, environmental extrapolation of the purported “systemic effects” of NPs should be approached with caution [239]. On the one hand, it is important to clearly distinguish evidence derived from environmental monitoring from results obtained in laboratory exposure studies [246]. On the other hand, conclusions are more appropriately framed in terms of “possible” or “potential” effects, with explicit acknowledgment of evidentiary boundaries, to avoid inferences that exceed current in situ detection capabilities. Future progress in this area will depend less on generating additional isolated findings than on improving reproducibility and comparability across studies, for example through the development of standardized analytical workflows and robust quality assurance/quality control (QA/QC) frameworks, including consistent reporting of size fractions, blanks and recovery rates, detection limits, and associated uncertainties [246]. Only with such foundational improvements can uncertainty in exposure assessment be reduced and the reliability of ecological risk evaluations be meaningfully strengthened.

**Table 1:** Composition of biofilm communities attached to marine MNPs.

Microorganism	Plastic Type	Plastic Size	Reference
Gammaproteobacteria, Actinobacteria, Alphaproteobacteria	LDPE, PS, PP	1–3 mm	[249]
Alphaproteobacteria, Gammaproteobacteria, Bacteroidetes	PE, PP, PS	0.3–5 mm	[250]
Gammaproteobacteria, Betaproteobacteria, Alphaproteobacteria	PE, PET	3–5 mm	[251]
Flavobacteriia, Alphaproteobacteria, Betaproteobacteria	HDPE, PS	3 mm	[252]
Gammaproteobacteria Alphaproteobacteria, Bacteroidia	PE, PP, PS	3–5 mm	[253]
Alphaproteobacteria, Gammaproteobacteria	PE, PVC	3.5–4.0 mm	[254]
Actinomycetia, Alphaproteobacteria, Betaproteobacteria	PE, PP, PS	2–2.5 mm	[255]

## 7. Risks of MNPs to the Environment and Ecosystems

### 7.1. Ecological Risks of MNPs in Typical Ecosystems: A Case Study of Coral Reefs and Seagrass Beds

MNPs, due to their nanoscale size, high surface-area-to-volume ratio, and reactivity, are of increasing ecological concern in tropical marine ecosystems [256]. Coral reefs and seagrass meadows are known for their structural complexity and sediment retention capacity. They serve as major MNP sinks and hotspots for pollutant accumulation and trophic transfer [257]. In coral reefs, MNPs have been detected in sediments, soft tissues, and calcium carbonate skeletons [258]. They can be directly taken up or transferred via trophic interactions. This can lead to oxidative stress, altered gene expression, tissue damage, apoptosis, and coral bleaching [259]. Coral responses are highly species-specific. They are modulated by morphology, feeding behavior, and particle properties [260]. Particle traits influence whether plastics are entrapped in mucus and subsequently ingested [261]. Environmental forces such as currents and colony structure also affect deposition [262]. Similarly, seagrass meadows in shallow coastal areas trap large quantities of MNPs in sediments and on blade surfaces. Epiphyte-associated immobilization can further enhance contaminant transfer to primary producers [263]. Plastics that are adhered to seagrass blades can impair light penetration and nutrient flow [264]. They can also elevate localized toxicity [264]. In some cases, however, plastics may suppress epiphyte overgrowth and facilitate carbon acquisition [265]. These interactions reveal the dual ecological impacts of MNPs.

MNPs disrupt photosynthesis, respiration, and energy metabolism in both corals and seagrasses. They often induce oxidative stress and tissue-level damage. High-dose exposures impair root integrity and shoot regeneration in *Cymodocea nodosa* [266]. They also reduce photosynthetic performance in *Zostera marina* [267]. This reduction may be driven by leachate toxicity [267]. In corals, MNPs affect predation and feeding behavior [268]. They also influence detoxification and symbiont function [268]. Colonization by Plastisphere microbes may disrupt coral microbiota [269]. This disruption can increase infection risks [269]. Pollutant-adsorbing MNPs facilitate contaminant transfer into food webs. This transfer can occur via ingestion or sedimentation [270]. In Bintan Island seagrass systems, high MP burdens have been recorded in benthic invertebrates. This pattern suggests trophic transfer [271]. Herbicide-laden MNPs exhibit synergistic phytotoxicity in *Halophila ovalis* [272]. Climate stressors exacerbate these impacts. Plastic exposure raises coral disease risk up to 22-fold [273]. Heat stress also enhances MNP uptake and retention in bleached corals [274]. Under elevated CO_2_ and variable light, MNPs disrupt metabolic processes in primary producers. The combined effects of MNPs, pollutants, and climate change threaten the structural and functional stability of coral reefs and seagrass ecosystems.

### 7.2. Interaction Between MNPs and Chemical Pollutants

Recent studies highlight the central role of MNPs in mediating the transport and bioavailability of environmental pollutants [275]. MNPs are not only passive reservoirs but also active carriers that amplify ecological risks through strong surface reactivity and high affinity for organic contaminants. Acting as “Trojan horses”, MNPs facilitate the accumulation and internal delivery of pollutants, driven by colloidal-level interactions and polymer-specific physicochemical traits [276]. On this basis, a growing body of evidence suggests that MPs and NPs, when acting as carriers of environmental contaminants, may increase the bioavailability of certain known or potential carcinogenic chemicals, thereby raising concerns about potential human health risks, including carcinogenic effects [277]. Co-exposure to MNPs and contaminants often results in synergistic or additive toxicities. For example, combined exposure to LDPE and benzo[a]pyrene (BaP) increases oxidative stress in bivalves, impairs lysosomal stability, reduces immune capacity, and alters immune cell ratios [278]. Similarly, co-exposure to 500 nm plastics with BaP and estradiol (E2) causes immune cell structural damage; suppresses phagocytic activity; disrupts ROS, Ca^2+^, and lysozyme levels; and downregulates immune-related genes [279]. These effects also occur with larger particles (30 μm), emphasizing the broad size range of MNPs contributing to immunotoxicity.

Environmental aging of plastic particles can further alter their toxicological behavior. For instance, co-exposure to triclosan and aged PE-MPs enhances pollutant accumulation and antioxidant disruption in mussels [280]. Synergistic toxicity may reflect not only MNPs’ vector role, but also pollutant bioavailability and cumulative chemical burdens [281]. In some cases, MNPs exhibit antagonistic effects by reducing contaminant bioavailability, even conferring partial protective effects against antibiotics and metals through adsorption [282]. Furthermore, MNPs can interfere with detoxification by impairing filtration rates and efflux transporters like P-glycoprotein, thereby limiting contaminant elimination [283]. Environmental conditions such as pH also influence pollutant release. Under acidic gastrointestinal conditions, desorption of persistent organic pollutants from plastics can increase up to 30-fold relative to seawater, enhancing internal exposure and toxicity [284].

Given the high diversity of MNP–contaminant interactions, different combinations vary substantially in their likelihood of environmental co-occurrence, exposure pathways, and potential ecological consequences [102]. As a result, discussions of combined effects that are limited to listing possible pairings risk weakening environmental relevance and interpretability. A more informative approach is therefore to adopt a scenario-based perspective, building on mechanistic understanding to focus on combinations that are more likely to occur in the environment and to be ecologically meaningful [240]. In this review, MNP–contaminant combinations of higher ecological relevance are considered to be those that (i) are more likely to co-occur in nearshore areas or regions with strong anthropogenic inputs, (ii) involve adsorption–desorption processes that can more readily translate into biologically available co-exposures under environmental or physiological conditions, and (iii) include contaminants with well-established toxicological properties or existing evidence of combined effects [285].

Based on the current literature, two representative scenarios warrant particular attention. One involves interactions between non-polar plastics (e.g., PE and PP) and hydrophobic organic contaminants (e.g., PAHs and PCBs), which are frequently reported to co-occur in the environment and whose adsorption behavior and potential carrier effects are relatively well characterized [286]. The other concerns interactions between environmentally aged or artificially aged plastics and polar contaminants such as metal ions and antibiotics, where changes in surface properties are more likely to alter contaminant binding and bioavailability. In addition, contaminants with endocrine-disrupting properties, which are highly sensitive during reproductive and developmental processes, merit increased consideration under long-term or chronic exposure scenarios, even when their environmental concentrations are relatively low [287].

### 7.3. Disruption of Ecosystem Nutrient Cycling and Primary Productivity by MNPs

MNPs are increasingly recognized as active agents influencing global biogeochemical cycles, particularly carbon cycling [288] (Table 2). In aquatic systems, MNPs interfere with carbon fixation by inhibiting phytoplankton growth and reducing chlorophyll a content, thereby weakening the oceanic carbon sink capacity [289]. Similar disruptions are observed in terrestrial plants, where MNPs reduce leaf area, enzyme activity, and nutrient uptake [290]. MNPs may further disrupt carbon cycling by altering functional enzyme activities and gene expression related to carbon metabolism [291]. Some plastic-derived carbon is metabolized by specific microbial taxa, promoting anaerobic processes and accelerating nutrient loss in sediments [292]. Biodegradable plastics stimulate specific microbial communities and enhance carbon turnover [293], and by 2025, plastic-derived carbon may contribute up to 6.8% of sedimentary organic carbon. MNPs alter microbial diversity and functional capacity, increasing CO_2_ emissions, dissolved organic carbon levels, and microbial metabolic rates [294]. They can also reshape carbon processing through enhanced microbial degradation of organic substrates and altered biofilm formation, buoyancy, and stress adaptability [295].

In coastal ecosystems, MNPs negatively affect plant growth and net primary productivity (NPP), particularly in seagrasses and epiphytic algae. MPs inhibit photosynthesis, respiration, and symbiotic microbial function in seagrasses, with long-term exposure reducing carbon fixation efficiency [296]. Their hydrophobic surfaces also adsorb heavy metals and organic pollutants, indirectly compromising plant health [297]. While epiphytic algae can mitigate toxicity via pollutant adsorption, they are sensitive to photosynthetic inhibition and mechanical damage from larger particles [298]. MPs impair nitrogen cycling, rhizospheric fixation, and sediment structure, disrupting nutrient uptake and reducing NPP [299]. They also alter shoot-to-root ratios, affecting carbon allocation [300]. Of particular concern is the translocation of NPs from roots to shoots, inducing physiological toxicity and reducing root function [300]. Although some plants may exhibit ecological adaptation, the chronic accumulation of MNPs poses long-term risks to carbon sink stability [301].

Based on available studies, measurements and modeling efforts have begun to target key processes through which MNPs may influence carbon sequestration, including particle aggregation and marine snow formation, changes in sinking and settling velocities, co-aggregation with minerals or organic matter, and the resulting effects on particle density and transport efficiency, as well as fecal pellet production associated with food web processes [302]. These studies provide valuable quantitative insights into the process-level mechanisms governing carbon export to the deep ocean [112]. However, comparability among reported results remains limited, largely due to differences in particle properties (e.g., polymer type, morphology, and degree of aging), background particle regimes, and conditioning by dissolved organic matter or biofilms, as well as inconsistencies in the definition of exposure metrics and flux-related endpoints [303].

It should also be noted that the influence of MNPs on carbon sequestration is likely to be strongly environment-dependent and not unidirectional. Under certain conditions, “ballasting” or carrier effects may enhance particle aggregation and increase the efficiency of downward carbon export [304]. In other contexts, however, MNPs may suppress primary production, alter microbial remineralization processes, or reduce effective carbon export through food web pathways, thereby weakening the biological pump [305]. Because these opposing effects may differ in direction and magnitude across regions with contrasting background particle concentrations, nutrient regimes, ecosystem structures, and particle type assemblages—and may even offset one another—current evidence remains insufficient to reliably quantify a net effect on carbon sequestration using a unified framework [306].

### 7.4. Scenario-Based Ecological Risk Assessment of Marine MNPs

It should be noted that when addressing the ecological risks of marine MNPs, risk assessment can be usefully structured into three sequential components: exposure assessment, effect assessment, and risk characterization. Specifically, predicted or measured environmental concentrations (PECs) are first determined across different environmental media and spatial scenarios; effect thresholds derived from laboratory toxicity data are then integrated using species sensitivity distributions (SSDs) to derive predicted no-effect concentrations (PNECs), typically expressed as HC5 values and adjusted using appropriate assessment factors (AFs); finally, risks are compared across scenarios using the risk quotient (RQ = PEC/PNEC) [307]. Using the quantitative example provided by Besseling et al. [307], SSDs constructed from multispecies chronic thresholds yielded an HC5 for microplastics in the water column in the order of 10^2^–10^3^ particles L^−1^ (particle-number-based), which was further translated into a conservative screening benchmark through the application of AFs. Comparing such thresholds with exposure levels across environmental scenarios is central to scenario-based risk evaluation. Available results indicate that worst-case MP concentrations in nearshore surface waters approach the same order of magnitude as the HC5, whereas corresponding worst-case concentrations in freshwater and open ocean surface waters are typically several orders of magnitude lower, suggesting that potential risks are more likely to occur in nearshore or accumulation-prone hotspot regions. It should be emphasized, however, that uncertainties in exposure estimates can substantially influence these interpretations; for example, reliance on surface-only sampling may systematically underestimate water column concentrations, thereby increasing uncertainty in nearshore risk characterization [308]. In contrast, scenario-based quantitative risk assessment for NPs is currently subject to more stringent data limitations. Although experimental effect thresholds have been used to derive HC5 values for NPs (particle-number-based), and these thresholds are generally several orders of magnitude higher than those reported for microplastics, empirical measurements of environmental NPs’ particle number concentrations remain scarce. This lack of exposure data limits the feasibility of direct PEC–PNEC comparisons and RQ calculations under present conditions [309]. In this context, some studies have employed order-of-magnitude projections based on fragmentation processes, suggesting that continued breakdown of larger plastic debris could substantially increase environmental NP abundances over long timescales. Such considerations shift the focus of risk discussion from currently verifiable quantitative assessments toward longer-term trends and uncertainty management. Accordingly, a cautious and proportionate approach to risk assessment is warranted. For microplastics, scenario-based comparisons should prioritize nearshore, estuarine, and other high-exposure environments and should report the orders of magnitude of PECs, PNECs, and RQs where data permit. For nanoplastics, it is important to explicitly acknowledge current gaps in environmental exposure data and methodological constraints and frame conclusions in terms of potential risks or trend-based inferences rather than extrapolating laboratory effects directly to environmentally substantiated systemic risks [307].

**Table 2:** Impacts of MNPs on the environmental carbon cycle.

Environment	Plastic Type	Effects	Reference
Water	PS	Inhibits the growth and chlorophyll synthesis of Scenedesmus obliquus, resulting in a reduction in Daphnia body size.	[310]
	PS	Inhibits the growth of *Microcystis aeruginosa* and alters the expression of toxin-related genes.	[311]
	Nylon MPs	Suppresses the growth of *Microcystis aeruginosa* and disrupts its photosynthetic activity and carbon fixation mechanisms.	[312]
	PS	Reduces the accumulation of photosynthetic pigments in Chlorella pyrenoidosa and damages its cell membrane structure, thereby impairing photosynthetic efficiency and cellular stability.	[313]
Sediment	PET	Elevates total organic carbon (TOC) levels in sediments and significantly alters microbial community structure and diversity.	[314]
	PE	Reduces carbon and nitrogen contents in Vallisneria natans leaves and enhances TOC accumulation in sediments.	[315]
	Degradable plastic	Increases the sediment carbon-to-nitrogen (C/N) ratio, thereby promoting anaerobic metabolic pathways.	[292]

## 8. Knowledge Gaps and Mitigation Priorities

### 8.1. Recycling as Both a Mitigation Pathway and a Potential Source of MNPs

From a life cycle management perspective, improving plastic recycling rates and material circularity is commonly regarded as an important pathway for reducing plastic inputs into the environment. Multiple policy analyses and scenario studies indicate that when interventions span key stages of plastic’s life cycle—including production, use, and end-of-life management—and explicitly incorporate recycling systems, the overall risk of plastic leakage to the environment can be substantially reduced [316]. However, a growing body of evidence also suggests that recycling processes are not entirely “emission-free,” and that their potential for secondary particle release warrants careful consideration [33,317]. Empirical measurements have detected high abundances of MPs in recycling wash water from mixed-plastic recovery facilities, indicating that recycling operations may, under certain conditions, represent previously underestimated point sources [33]. In parallel, global-scale modeling studies predict that if recycling capacity continues to expand without corresponding control measures, MP emissions associated with recycling activities could increase in the future [318].

In this context, translating recycling into an effective mitigation strategy likely requires not only system-level expansion but also strengthened operational management and end-of-pipe controls at recycling facilities [33]. For example, enhancing filtration, sedimentation, and membrane treatment of recycling wash water, together with the implementation of closed-loop water circulation where feasible, could reduce the risk of particle release via effluent discharge. In addition, the installation of dust capture and particulate control systems for crushing, conveying, and related unit operations would help limit airborne transport and associated occupational exposure. Future research is therefore needed to systematically quantify MNP generation and removal efficiencies per unit throughput across different recycling technologies and operating conditions, with the aim of establishing comparable emission factors that can support regulatory assessment and evidence-based management decisions [317].

### 8.2. Environmental Realism and Inconsistencies

In studies of MNPs, discrepancies among reported conclusions largely arise not from inherent biological variability, but from limited environmental realism and poor comparability across studies [246]. Although many investigations nominally address the same “microplastic exposure,” differences in particle properties, exposure scenarios, and quantification metrics often prevent direct comparison of effect direction and magnitude [5]. Among these factors, particle size spectra and morphology represent the most common and critical sources of divergence [319]. For experimental controllability, laboratory studies typically employ commercially available microspheres with narrow size distributions and regular shapes, whereas MNPs in marine environments occur as continuous, broad size spectra and coexist as fibers, fragments, and films [319]. Because particle size and morphology jointly influence aggregation and settling behavior, ingestion probability, gastrointestinal residence time, and the potential for translocation across tissues, even modest differences in size windows or morphotypes can lead to apparently contradictory outcomes across studies [320].

Beyond physical characteristics, differences in polymer type, additive composition, and aging state further amplify inter-study incomparability [321]. Polymers such as PE, PP, PET, PVC, and PA differ intrinsically in density, surface properties, and sorption behavior, while environmentally derived particles frequently carry leachable additives or processing residues, resulting in more complex chemical exposure contexts [5]. Moreover, aging processes are simulated using diverse pathways and intensities across studies; consequently, even identical polymers may exhibit non-equivalent surface chemistries under different experimental conditions [39]. Methodological variability also plays a substantial role: particle isolation and digestion procedures can introduce size-selective biases, and differences among identification and quantification techniques—each with distinct size ranges and measurement logics—hinder harmonization of exposure metrics. In the absence of essential conversion parameters, such as particle size distributions, density, or aggregation state, cross-study comparisons often remain numerical rather than dose-equivalent [322]. Additionally, the frequent use of single-factor, high-concentration, or short-term exposures contrasts sharply with the long-term, low-dose, and multi-stressor conditions that are characteristic of marine environments, further increasing uncertainty in environmental extrapolation [321]. Accordingly, reducing inconsistencies in the literature requires not the pursuit of a single “environmentally relevant” concentration, but rather improved environmental representativeness of particle properties, transparent and standardized quantification frameworks, and sufficient replication coupled with validated exposure characterization to enhance comparability across studies [321].

## 9. Conclusions

As emerging contaminants, MNPs exhibit complex physicochemical properties and dynamic transport behaviors, leading to their pervasive distribution in seawater, sediments, and marine biota. Their high bioavailability across diverse marine taxa enables ingestion and subsequent physiological disturbances, including oxidative stress, metabolic disruption, immune suppression, and tissue accumulation, which collectively threaten organismal health and influence population dynamics. Beyond direct toxicity, MNPs exert broader ecological impacts through trophic transfer, microbial colonization via the Plastisphere, and synergistic interactions with coexisting pollutants. In vulnerable ecosystems such as coral reefs, mangroves, and seagrass meadows, MNPs can impair photosynthesis, alter nutrient and carbon cycling, and accelerate ecological degradation, thus increasing system vulnerability. Although advances have been made in understanding degradation processes, including biodegradation, photodegradation, and oxidative breakdown, critical knowledge gaps remain concerning the long-term fate of MNPs, their fine-scale ecological effects, and interactions with global stressors such as ocean warming and acidification. Technical barriers also persist in the detection, quantification, and toxicity evaluation of MNPs. Addressing these challenges requires integrated, interdisciplinary research efforts. Future priorities should focus on standardized detection methodologies, comprehensive assessments of ecosystem-level responses, and mechanistic studies in toxicology and ecological risk analysis. Such coordinated approaches are essential to inform science-based management and policy interventions aimed at mitigating the impacts of plastic pollution in marine environments.

## Data Availability

No new data were created or analyzed in this study. Data sharing is not applicable to this article.

## Supplementary Materials

The following supporting information can be downloaded at https://www.mdpi.com/article/10.3390/toxics14020120/s1: Figure S1. Keyword co-occurrence network of included literature. Table S1: Marine bacteria capable of degrading MNPs. Table S2: The degradative role of marine fungi on MNPs. Table S3: Microbial enzymes capable of degrading plastics. Table S4. Representative field and mesocosm evidence for the enrichment, fate, and ecological effects of MNPs in marine environments. References [106,107,108,110,111,152,153,258,259,260,262,263,266,267,323,324,325,326,327,328,329,330,331,332,333,334,335,336,337,338,339,340,341,342] are cited in the Supplementary Materials.
