# Supplementary material for: From Enrichment to Fate: Transport, Transformation, and Fate of Micro- and Nanoplastics in Marine Environments
Source: Toxics. 2026 Jan 27;14(2):120. doi: 10.3390/toxics14020120 (PMC12944654; doi:10.3390/toxics14020120)
Supplement: Supplementary file 1 [file toxics-14-00120-s001.zip › toxics-4088120-supplementary.pdf]

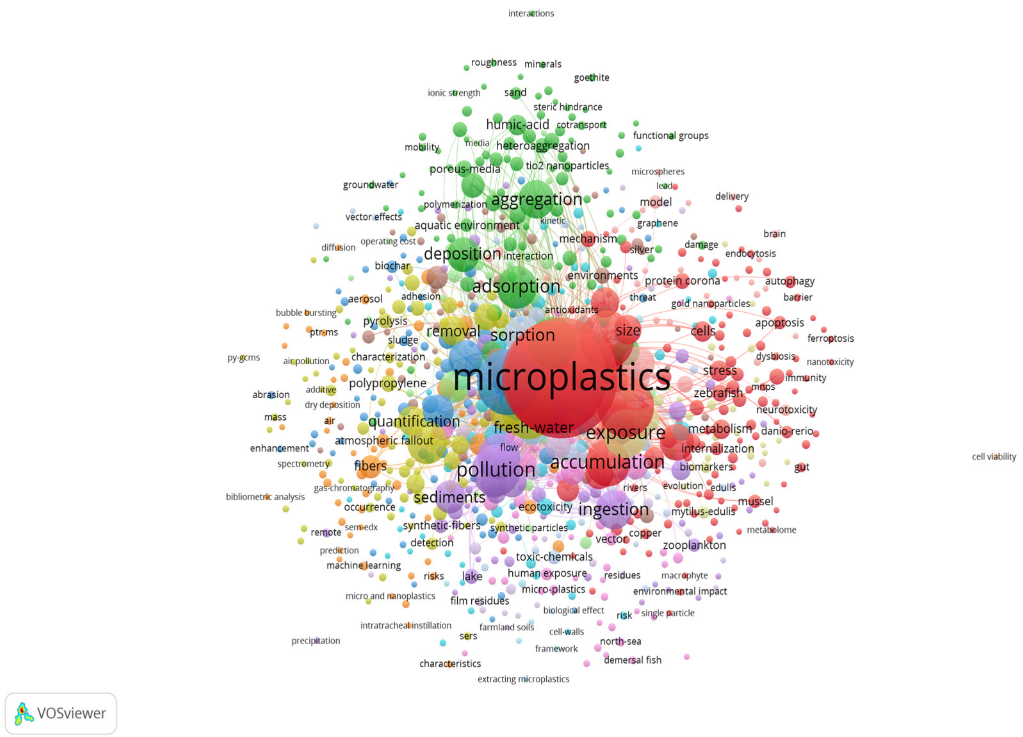

Figure S1. The literature co-occurrence network diagram generated using VOSviewer® software represents each keyword as a circle, with the size of the circle corresponding to the frequency with which that keyword co-occurs in the literature. The colors in the network visualization indicate the cluster category to which each keyword belongs, focusing on sources and accumulation of MNPs in the marine environment.

Table S1. Marine bacteria capable of degrading MNPs.

Microorganism	Plastic type	Reference
Vibrio parahaemolyticus, Shewanella indica	LDPE powder	[1]
Pseudomonas pertucinogena	PCL, PU emulsion	[2]
Entrobacter sp., Bacillus sp.,	PHA powder,	[3]
Pseudomonas, Alcanivorax, Tenacibaculum	PCL granule	[4]
Psychrobacter sp., Pseudomonas sp.,	PCL fibers	[5]
Vibrio alginolyticus, Vibrio parahaemolyticus	PVA	[6]
Bacillus cereus, Bacillus gottheilii	PE, PET, and PS-MPs	[7]
Bacillus sp. Rhodococcus sp.	PP-MPs	[8]

Table S2. The degradative role of marine fungi for MNPs.

**Microorganism**	**Plastics type**	**Reference**
Asteromyces cruciatus, Candida guillermondii	PHAs	[9]
Phaeophleospora eucalypticola, Cladosporium xanthochromaticum	PCL	[10]
Aspergillus versicolor, Aspergillus sp.	LDPE	[11]
Aspergillus niger, Aspergillus flavus	LDPE	[12]
Zalerion maritimum	PE	[13]
Fusarium oxysporum	Polyester fibers	[14]

Table S3. Microbial enzymes capable of degrading plastics.

Microorganism	Enzyme	Plastic	Reference
Arthrobacter sp.	Hydrolase	LDPE	[15]
Acinetobacter sp.	Alkane hydroxylase and alcohol dehydrogenase	PE	[16]
Exiguobacterium sp.	Laccase and Alkane Hydroxylase	LDPE	[17]
Pseudomonas sp.	alkB1 and alkB2 gene	PE	[18]
Rhodococcus sp.	Hydrolase	PP	[8]
Lysinibacillus sp.	Depolymerase	PE、PP	[19]
Ideonella sp.	PETase, MHETase and hydrolase	PE	[20]

Table S4. Representative field and mesocosm evidence for the enrichment, fate, and ecological effects of MNPs in marine environments.

Study type	Medium / Matrix	MNP size and polymer	Endpoint	Key takeaway	Ref
Field (in situ seafloor survey)	Seafloor / deep-sea environment	MPs	Enrichment / hotspot formation	Demonstrates that deep-sea circulation can create “seafloor hotspots” of microplastic accumulation, supporting a real-ocean physical control on final sinks.	[21]
Field (water column observations)	Ocean water (subsurface)	MPs	Hidden inventory / vertical distribution	Reports high concentrations of plastics beneath the surface, highlighting that surface	[22]

Study type	Medium / Matrix	MNP size and polymer	Endpoint	Key takeaway	Ref
				measurements alone can underestimate real-ocean inventories.	
Field synthesis (multiple sites)	Deep sea / seabed	MPs	Final sink	Provides evidence that the deep sea acts as a major sink for microplastic debris, reinforcing the pelagic-to-benthic transfer in real systems.	[23]
Process-focused (field-informed)	Deep-marine sediments	MPs	Transport and burial	Shows that turbidity currents can transport and bury microplastics in deep-marine sediments, supporting episodic events as a burial mechanism.	[24]
Global estimate (model/compilation)	Seafloor reservoir	MPs	Magnitude of sink	Provides a global estimate of the ocean floor plastic reservoir, helping constrain the scale of deep-sea accumulation.	[25]
Field (coastal surveys)	Seagrass beds / coastal environment	MPs	Enrichment / trapping	Identifies seagrass beds as an emerging coastal hotspot that traps microplastics, supporting habitat-mediated retention in real coastal systems.	[26]

Study type	Medium / Matrix	MNP size and polymer	Endpoint	Key takeaway	Ref
Field (coastal trapping)	Seagrass meadows / shoreline interface	Litter/MPs	Retention / interception	Demonstrates the role of seagrass meadows in coastal trapping of litter (including plastics), supporting ecosystem-scale interception processes.	[27]
Mesocosm / exposure-bridging	Seagrass physiology	MPs/NPs	Effect (growth/physiology)	Provides early evidence that MP/NP exposure can impact seagrass growth and physiology under controlled but ecologically relevant designs.	[28]
Field-linked / exposure study	Seagrass + epiphytes	MPs	Effect (respiration)	Reports an association between microplastic pollution and reduced respiration in seagrass and epiphytes, suggesting potential functional impacts at the habitat level.	[29]
Field / case study	Coral reef system (Palau)	Plastics	Threat characterization	Case evidence that plastics represent a threat in a real coral reef setting, supporting reef vulnerability under field conditions.	[30]
Field + lab / mixed evidence	Scleractinian corals	MPs	Effect (coral health)	Reports impacts on nearshore	[31]

Study type	Medium / Matrix	MNP size and polymer	Endpoint	Key takeaway	Ref
				scleractinian corals in a real reef region, bridging environmental occurrence with biological responses.	
Field (biota contamination)	Coral tissue	Microfibers	Enrichment in biota	Shows that microfiber abundance in coral tissues varies geographically, providing field evidence for spatially heterogeneous biological burdens.	[32]
Process-informed (mechanistic, marine-relevant)	Coral surface	MPs	Habitat sink process	Proposes/quantifies adhesion to coral surfaces as a potential sink for marine microplastics, supporting habitat-mediated retention mechanisms.	[33]
Field-informed synthesis	Marine habitat-formers	MPs	Multiple impacts / risk	Synthesizes evidence that microplastics can threaten marine habitat-forming species, supporting ecosystem relevance beyond single-species lab tests.	[34]

References

1. Joshi, G., Goswami, P., Verma, P., Prakash, G., Simon, P., Vinithkumar, N.V., Dharani, G. Unraveling the plastic degradation potentials of the plastisphere-associated marine bacterial consortium as a key player for the low-density polyethylene degradation. J. Hazard. Mater. 2022, 425, doi.org/10.1016/j.jhazmat.2021.128005.
2. Molitor, R., Bollinger, A., Kubicki, S., Loeschcke, A., Jaeger, K.E., Thies, S. Agar plate-based screening methods for the identification of polyester hydrolysis by Pseudomonas species. Microb. Biotechnol. 2020, 13, 274-284, doi.org/10.1111/1751-7915.13418.
3. Volova, T., Boyandin, A., Vasiliev, A., Karpov, V., Prudnikova, S., Mishukova, O., Boyarskikh, U., Filipenko, M., Rudnev, V., Xuân, B.B. Biodegradation of polyhydroxyalkanoates (PHAs) in tropical coastal waters and identification of PHA-degrading bacteria. Polym. Degrad. Stab. 2010, 95, 2350-2359, doi.org/10.1016/j.polymdegradstab.2010.08.023.
4. Sekiguchi, T., Saika, A., Nomura, K., Watanabe, T., Watanabe, T., Fujimoto, Y., Enoki, M., Sato, T., Kato, C., Kanehiro, H. Biodegradation of aliphatic polyesters soaked in deep seawaters and isolation of poly (ε-caprolactone)-degrading bacteria. Polym. Degrad. Stab. 2011, 96, 1397-1403, doi.org/10.1016/j.polymdegradstab.2011.03.004.
5. Sekiguchi, T., Sato, T., Enoki, M., Kanehiro, H., Uematsu, K., Kato, C. Isolation and characterization of biodegradable plastic degrading bacteria from deep-sea environments. JAMSTEC Report of Research Development. 2011, 11, 33-41, doi.org/10.5918/jamstecr.11.33.
6. Raghul, S.S., Bhat, S.G., Chandrasekaran, M., Francis, V., Thachil, E.T. Biodegradation of polyvinyl alcohol-low linear density polyethylene-blended plastic film by consortium of marine benthic vibrios. Int. J. Environ. Sci. Technol. 2014, 11, 1827-1834, doi.org/10.1007/s13762-013-0335-8.
7. Auta, H.S., Emenike, C.U., Fauziah, S.H. Screening of Bacillus strains isolated from mangrove ecosystems in Peninsular Malaysia for microplastic degradation. Environ. Pollut. 2017, 231, 1552-1559, doi.org/10.1016/j.envpol.2017.09.043.
8. Auta, H.S., Emenike, C.U., Jayanthi, B., Fauziah, S.H. Growth kinetics and biodeterioration of polypropylene microplastics by Bacillus sp and Rhodococcus sp isolated from mangrove sediment. Mar. Pollut. Bull. 2018, 127, 15-21, doi.org/10.1016/j.marpolbul.2017.11.036.
9. Matavulj, M., Molitoris, H.P. Marine fungi: degraders of poly-3-hydroxyalkanoate based plastic materials. Zbornik Matice srpske za prirodne nauke. 2009, 253-265, doi.org/10.2298/ZMSPN0916253M.
10. Kim, S.H., Lee, J.W., Kim, J.S., Lee, W., Park, M.S., Lim, Y.W. Plastic-inhabiting fungi in marine environments and PCL degradation activity. Anton Leeuw Int J G. 2022, 115, 1379-1392, doi.org/10.1007/s10482-022-01782-0.
11. Pramila, R., Ramesh, K.V. Biodegradation of low density polyethylene (LDPE) by fungi isolated from marine water a SEM analysis. Afr J Microbiol Res. 2011, 5, 5013-5018.
12. Ameen, F., Moslem, M., Hadi, S., Al-Sabri, A.E. Biodegradation of Low Density Polyethylene (LDPE) by Mangrove Fungi From the Red Sea Coast. Prog Rubber Plast Re. 2015, 31, 125-143, doi.org/10.1177/147776061503100204.
13. Paço, A., Duarte, K., da Costa, J.P., Santos, P.S.M., Pereira, R., Pereira, M.E., Freitas, A.C., Duarte, A.C., Rocha-Santos, T.A.P. Biodegradation of polyethylene microplastics by the marine fungus

Zalerion maritimum. Sci. Total Environ. 2017, 586, 10-15, doi.org/10.1016/j.scitotenv.2017.02.017.

14. Taniguchi, I., Yoshida, S., Hiraga, K., Miyamoto, K., Kimura, Y., Oda, K. Biodegradation of PET: Current Status and Application Aspects. Acs Catalysis. 2019, 9, 4089-4105, doi.org/10.1021/acscatal.8b05171.
15. Pereira, C., Kingsley, S.J., Savariar, V. Biodegradation of Pre-Treated Low Density Polyethylene Spent Saline Vials by selected Arthrobacter Spp. The Journal of Solid Waste Technology Management. 2012, 38, 225-231, doi.org/10.5276/jswtm.2012.225.
16. Kim, H.R., Lee, C., Shin, H., Kim, J., Jeong, M., Choi, D. Isolation of a polyethylene-degrading bacterium, Acinetobacter guillouiae, using a novel screening method based on a redox indicator. Heliyon. 2023, 9, doi.org/10.1016/j.heliyon.2023.e15731.
17. Maroof, L., Khan, I., Hassan, H., Azam, S., Khan, W. Microbial degradation of low density polyethylene by Exiguobacterium sp. strain LM-IK2 isolated from plastic dumped soil. WORLD J MICROB BIOT. 2022, 38, doi.org/10.1007/s11274-022-03389-z.
18. Jeon, H.J., Kim, M.N. Isolation of mesophilic bacterium for biodegradation of polypropylene. Int. Biodeterior. Biodegrad. 2016, 115, 244-249, doi.org/10.1016/j.ibiod.2016.08.025.
19. Jeon, J.M., Park, S.J., Choi, T.R., Park, J.H., Yang, Y.H., Yoon, J.J. Biodegradation of polyethylene and polypropylene by Lysinibacillus species JJY0216 isolated from soil grove. Polym Degrad Stabil. 2021, 191, doi.org/10.1016/j.polymdegradstab.2021.109662.
20. Yoshida, S., Hiraga, K., Takehana, T., Taniguchi, I., Yamaji, H., Maeda, Y., Toyohara, K., Miyamoto, K., Kimura, Y., Oda, K. A bacterium that degrades and assimilates poly(ethylene terephthalate). Science. 2016, 351, 1196-1199, doi.org/10.1126/science.aad6359.
21. Kane, I.A., Clare, M.A., Miramontes, E., Wogelius, R., Rothwell, J.J., Garreau, P., Pohl, F. Seafloor microplastic hotspots controlled by deep-sea circulation. Science. 2020, 368, 1140-+, doi.org/10.1126/science.aba5899.
22. Pabortsava, K., Lampitt, R.S. High concentrations of plastic hidden beneath the surface of the Atlantic Ocean. Nat. Commun. 2020, 11, doi.org/10.1038/s41467-020-17932-9.
23. Woodall, L.C., Sanchez-Vidal, A., Canals, M., Paterson, G.L.J., Coppock, R., Sleight, V., Calafat, A., Rogers, A.D., Narayanaswamy, B.E., Thompson, R.C. The deep sea is a major sink for microplastic debris. Roy Soc Open Sci. 2014, 1, doi.org/10.1098/rsos.140317.
24. Pohl, F., Eggenhuisen, J.T., Kane, I.A., Clare, M.A. Transport and Burial of Microplastics in Deep-Marine Sediments by Turbidity Currents. Environ. Sci. Technol. 2020, 54, 4180-4189, doi.org/10.1021/acs.est.9b07527.
25. Zhu, X., Rochman, C.M., Hardesty, B.D., Wilcox, C. Plastics in the deep sea–A global estimate of the ocean floor reservoir. Deep Sea Research Part I: Oceanographic Research Papers. 2024, 206, 104266, doi.org/10.1016/j.dsr.2024.104266.
26. Huang, Y., Xiao, X., Xu, C., Perianen, Y.D., Hu, J., Holmer, M. Seagrass beds acting as a trap of microplastics-Emerging hotspot in the coastal region? Environ. Pollut. 2020, 257, 113450.
27. Navarrete-Fernández, T., Bermejo, R., Hernández, Deidun, A., Andreu-Cazenave, M., Cózar, A. The role of seagrass meadows in the coastal trapping of litter. Mar. Pollut. Bull. 2022, 174, doi.org/10.1016/j.marpolbul.2021.113299.
28. Menicagli, V., Castiglione, M.R., Balestri, E., Giorgetti, L., Bottega, S., Sorce, C., Spanò, C., Lardicci, C. Early evidence of the impacts of microplastic and nanoplastic pollution on the

growth and physiology of the seagrass Cymodocea nodosa. Sci. Total Environ. 2022, 838, 156514.

29. Molin, J.M., Groth-Andersen, W.E., Hansen, P.J., Kühl, M., Brodersen, K.E. Microplastic pollution associated with reduced respiration in seagrass (Zostera marina L.) and associated epiphytes. Front. Mar. Sci. 2023, 10, 1216299, doi.org/10.3389/fmars.2023.1216299.

30. Beraud, E., Bednarz, V., Otto, I., Golbuu, Y., Ferrier-Pages, C. Plastics are a new threat to Palau's coral reefs. Plos One. 2022, 17, doi.org/10.1371/journal.pone.0270237.

31. Lim, Y.C., Chen, C.W., Cheng, Y.R., Chen, C.F., Dong, C.D. Impacts of microplastics on scleractinian corals nearshore Liuqiu Island southwestern Taiwan. Environ. Pollut. 2022, 306, doi.org/10.1016/j.envpol.2022.119371.

32. Oldenburg, K.S., Urban-Rich, J., Castillo, K.D., Baumann, J.H. Microfiber abundance associated with coral tissue varies geographically on the Belize Mesoamerican Barrier Reef System. Mar. Pollut. Bull. 2021, 163, doi.org/10.1016/j.marpolbul.2020.111938.

33. Martin, C., Corona, E., Mahadik, G.A., Duarte, C.M. Adhesion to coral surface as a potential sink for marine microplastics. Environ. Pollut. 2019, 255, doi.org/10.1016/j.envpol.2019.113281.

34. Corinaldesi, C., Canensi, S., Dell'Anno, A., Tangherlini, M., Di Capua, I., Varrella, S., Willis, T.J., Cerrano, C., Danovaro, R. Multiple impacts of microplastics can threaten marine habitat-forming species. Commun Biol. 2021, 4, doi.org/10.1038/s42003-021-01961-1.
